# Potential effects of warmer worms and vectors on onchocerciasis transmission in West Africa

**DOI:** 10.1098/rstb.2013.0559

**Published:** 2015-04-05

**Authors:** Robert A. Cheke, Maria-Gloria Basáñez, Malorie Perry, Michael T. White, Rolf Garms, Emmanuel Obuobie, Poppy H. L. Lamberton, Stephen Young, Mike Y. Osei-Atweneboana, Joseph Intsiful, Mingwang Shen, Daniel A. Boakye, Michael D. Wilson

**Affiliations:** 1 Agriculture, Health and Environment Department, Natural Resources Institute, University of Greenwich at Medway, Central Avenue, Chatham Maritime, Kent ME4 4TB, UK; 2 Department of Infectious Disease Epidemiology, School of Public Health, Imperial College London, St Mary's Campus, Norfolk Place, London W2 1PG, UK; 3 Bernhard Nocht Institute for Tropical Medicine, Bernhard-Nocht-Strasse 74, Hamburg 20359, Germany; 4 Water Research Institute, Council for Scientific and Industrial Research, PO Box M32, Accra, Ghana; 5 Regional Institute for Population Studies, University of Ghana, PO Box LG 97, Legon, Accra, Ghana; 6 Department of Applied Mathematics, Xi'an Jiaotong University, Xi'an 710049, People's Republic of China; 7 Noguchi Memorial Institute for Medical Research, University of Ghana, PO Box LG 581, Legon, Accra, Ghana

**Keywords:** *Simulium damnosum* complex, *Onchocerca volvulus*, temperature, rainfall, river discharges, mathematical models

## Abstract

Development times of eggs, larvae and pupae of vectors of onchocerciasis (*Simulium* spp.) and of *Onchocerca volvulus* larvae within the adult females of the vectors decrease with increasing temperature. At and above 25°C, the parasite could reach its infective stage in less than 7 days when vectors could transmit after only two gonotrophic cycles. After incorporating exponential functions for vector development into a novel blackfly population model, it was predicted that fly numbers in Liberia and Ghana would peak at air temperatures of 29°C and 34°C, about 3°C and 7°C above current monthly averages, respectively; parous rates of forest flies (Liberia) would peak at 29°C and of savannah flies (Ghana) at 30°C. Small temperature increases (less than 2°C) might lead to changes in geographical distributions of different vector taxa. When the new model was linked to an existing framework for the population dynamics of onchocerciasis in humans and vectors, transmission rates and worm loads were projected to increase with temperature to at least 33°C. By contrast, analyses of field data on forest flies in Liberia and savannah flies in Ghana, in relation to regional climate change predictions, suggested, on the basis of simple regressions, that 13–41% decreases in fly numbers would be expected between the present and before 2040. Further research is needed to reconcile these conflicting conclusions.

## 1. Introduction

Vector-borne diseases such as malaria are likely to spread with climate change [1], but little attention has been paid to how future climatic regimes may affect onchocerciasis, a debilitating disease occurring in sub-Saharan Africa, Central and South America, and the Yemen. Onchocerciasis, or ‘river blindness’, owing to infection with the nematode parasite *Onchocerca volvulus* and transmitted by blackflies (*Simulium* spp.), causes visual impairment, blindness, a range of skin lesions and excess mortality [2,3]. It has been estimated that in Africa 37 million people were infected prior to the inception of the Onchocerciasis Control Programme in West Africa (OCP) and the African Programme for Onchocerciasis Control (APOC) [4,5]. In West Africa, the vectors are various cytoforms of the *Simulium damnosum* complex which differ in their ecologies [6] and vectorial roles [7]. Of the principal vectors in West Africa, *S. sanctipauli*, *S. soubrense* and *S. yahense* are found mostly in forests; *S. squamosum* principally occurs in highland zones, while *S. damnosum* s.str. and *S. sirbanum* are more widespread, but this latter pair are the only common species found in northern savannah zones. *Simulium soubrense, S. yahense*, *S. damnosum* s.str. and *S. sirbanum* occur in Liberia, the source of the forest data presented here; and at least six different cytospecies occur in Ghana [8], the source of the contrasting savannah data analysed in this paper. This diversity means that generalizations about effects of climate change on onchocerciasis transmission require caution unless the particular vector or vectors involved are specified, with similar caveats necessary for different river sizes and bioclimatic zones.

The immature stages (eggs, larvae and pupae) of blackflies (Diptera: Simuliidae) are found in fast flowing, highly oxygenated, water. Assuming an adequate food supply in unpolluted rivers, there is a variety of factors that determine: (i) the rate of development from egg to adult, which is principally governed by temperature; (ii) the densities of populations, which are affected by the development rates, river discharges, adult survival rates, immigration and emigration, and (iii) the geographical range, which depends on habitat type, temperature and river structure. In addition, fly size, fly fecundity and water quality will be influential in determining population densities. Generalizations about *S. damnosum* complex population dynamics are difficult given the importance of local conditions, particularly river topographies and the vegetation in and around river beds. Rising or falling river heights and discharges may either increase or decrease blackfly breeding opportunities. For instance, excellent breeding sites may disappear when a river floods or be created when previously dry rocks or vegetation become partially submerged leading to the formation of rapids. Furthermore, temperatures vary along rivers, increasing with distances from their sources and between neighbouring rivers [9].

The dependence of onchocerciasis vectors on temperature, rainfall and thus river discharges means that they will be affected by changes in climate. Here, we first examine empirical relationships on fly numbers and environmental variables. Next, we show the effects of temperature on vector development rates and survival, and on development of the parasite within the vector which is also temperature dependent [10,11]. We then developed a novel onchocerciasis vector population model to examine how changes in temperature affect fly numbers. The model was parametrized from data obtained from literature reviews and unpublished results. Much of the data came from four sites, two in Liberia and two in Ghana, for which climate change output from regional climate models (RCMs), downscaled from global climate change models, was linked to hydrological models for the relevant river basins. Finally, we linked the savannah vector model to a framework for the parasite's dynamics in humans and vectors [12] to illustrate how temperature may influence disease transmission through vector and parasite development and vector survival.

## 2. Material and methods

### (a) Literature review

A literature review identified publications that contained data on larval development of *O. volvulus* or survival and/or development of *Simulium* spp. at different temperatures. In addition to reviewing early and grey literature, electronic searches were conducted in 2013 using the databases PubMED and Web of Knowledge, supplemented by electronic searches of the DIALOG library conducted between the early 1980s and October 2000.

### (b) Data sources for environmental variables and onchocerciasis vectors in forest sites beside the St. Paul river, Liberia

Garms [13] studied the biology of *S. damnosum* s.l. in the Bong Range, a forested zone of Liberia, through which the St. Paul river flows. It is now known that the onchocerciasis vector that breeds in the St. Paul river, a member of the *S. sanctipauli* sub-complex, is *S. soubrense* [14], while vectors in some of its tributaries are *S. yahense* [15]. Here we will only consider populations of *S. soubrense*, which were never subject to larviciding, studied at two sites beside the St. Paul river: Haindi (6°53′45″ N, 10°22′48″ W), where data were collected weekly from October 1968 until December 1969 inclusive, and Gengema (6°54′6″ N, 10°21′44″ W), where data were collected monthly from February 1969 until February 1971 inclusive. Vector biting rates, parous rates and associated precipitation and temperature data were measured in the field or obtained from local sources.

### (c) Climate change predictions for the St. Paul river basin

For the Liberian climate projections, monthly and daily means of precipitation, temperature and potential evapotranspiration for the 1961–1990 period were obtained from the FAO New LocClim software and database [16]. Daily values of precipitation, minimum and maximum temperatures covering the period 1961–2040 were obtained from archives of the AMMA-EMSEMBLES ensemble-based runs for West Africa [17,18]. The period 1961–1990 was considered as the baseline period for this study, while the period 2011–2040 was considered as the period for the future scenario (denoted ‘2020s’). The New LocClim data were derived from observed data for Liberia and were used in this study as the observed data for the ‘baseline scenario’ as actual daily observed data for the baseline period were not available. The ensemble climate data used were from two RCMs (HadRM 3P and REMO) and were based on the IPCC SRES A1B scenario experiment. The HadRM 3P model was forced with the boundary conditions of the HadCM3 Global Climate Model (GCM) while REMO was forced with the boundary conditions of ECHAM5 GCM. The HadRM 3P and REMO models were chosen from 10 RCMs used in the AMMA-ENSEMBLES, because they represent the driest and wettest future climatic conditions that can be expected for the St. Paul river basin under the IPCC A1B scenario.

Biases in projections for the ‘2020s’ by the two RCMs were corrected using the ‘delta’ approach, which has been used extensively for corrections in RCM projections in climate change impact studies (e.g. [19,20]). The approach involved (i) computing monthly means of rainfall and temperature for the baseline period and the ‘2020s’ for the RCM data; (ii) determining future changes in precipitation and temperature by contrasting the monthly means for the ‘2020s’ with those of the baseline determined in (i), and (iii) applying the changes determined in (ii) to observed data to obtain the ‘2020s’ climate data for the impact studies.

The baseline and the ‘2020s’ climate data were used to drive Budyko's water balance model of a river basin [21] to predict likely changes in the ‘2020s’ compared with the baseline. The results showed that in the ‘2020s’, daily temperatures in the St. Paul river basin are expected to rise by 1.1–1.3°C, rainfall to either decrease by 3.6% (prediction based on HadRM 3P) or increase by 2% (prediction based on REMO) and the mean total annual river flow in the St. Paul to reduce by 0.7% (REMO) or 25% (HadRM 3P).

### (d) Data sources for environmental variables and onchocerciasis vectors in savannah sites of the Black Volta and Pru river basins, Ghana

Savannah regions of Ghana were part of the OCP, when rivers including the Black Volta and Pru were regularly treated with insecticides to kill the larvae of *S. damnosum* s.l. between 1975 and 2002 [22]. At the study sites used here, Agborle Kame (08°14′04″ N, 2°12′23″ W) on the Black Volta river and Asubende (08°01′01″ N, 00°58′54″ W) on the Pru river, the vectors are almost exclusively the savannah members of the *S. damnosum* complex, i.e. *S. damnosum* s.str. or *S. sirbanum* [8], for which historical OCP data on fly biting rates, parous rates and river discharges prior to larviciding treatments were available. Rainfall and temperature data were taken from the FAO New LocClim software and database [16].

### (e) Climate change predictions for the Black Volta and Pru river basins, Ghana

Daily observed data on rainfall and temperature for the period 1961–1990 for the Black Volta and Pru river basins were used as the ‘baseline scenario’ for determining changes in future climate and river flow in this study. The observed data were obtained mainly from the Ghana Meteorological Agency. For the Black Volta basin, climate data covering the part of the basin in Burkina Faso were obtained from the Direction de la Météorologie Nationale, Burkina Faso. The climate change scenarios used in this study were obtained from the ENSEMBLES project [17] for two RCMs (HadRM 3P and REMO) simulated under the IPCC A1B greenhouse gas emission experiment. The HadRM 3P and REMO models were driven by boundary conditions of the HadCM3 GCM and the ECHAM5 GCM, respectively. As for Liberia (§2c), the HadRM 3P and REMO models were chosen because they provided the two extremes of future projections from 10 RCMs over both the Black Volta and Pru basins. Data for both were obtained for the periods 1961–1990 (baseline) and 2011–2040 (‘2020s’). Biases in the RCM data were corrected using the ‘delta’ approach described in §2c.

The impact of changes in rainfall and temperatures on river flow in the Black Volta and Pru basins was assessed by integrating the climate change scenarios in the Soil and Water Assessment Tool (SWAT) hydrological model [23], which had been adapted to the two basins. Analysis of the mean of the climate data from HadRM 3P and REMO revealed that the mean daily temperature and annual total rainfall for the Black Volta basin will increase in the ‘2020s’ by 1.1°C and 3.1%, respectively, relative to the baseline. For the Pru basin, increases in the daily temperature and annual rainfall are projected to be 0.7°C and 2.3%, respectively. The mean annual river flow for the ‘2020s’, based on the mean of the climate data from the two RCMs, is expected to increase above the baseline value by 1.8% for the Pru basin and 0.8% for the Black Volta basin.

### (f) Water temperatures

**Figure 1.**
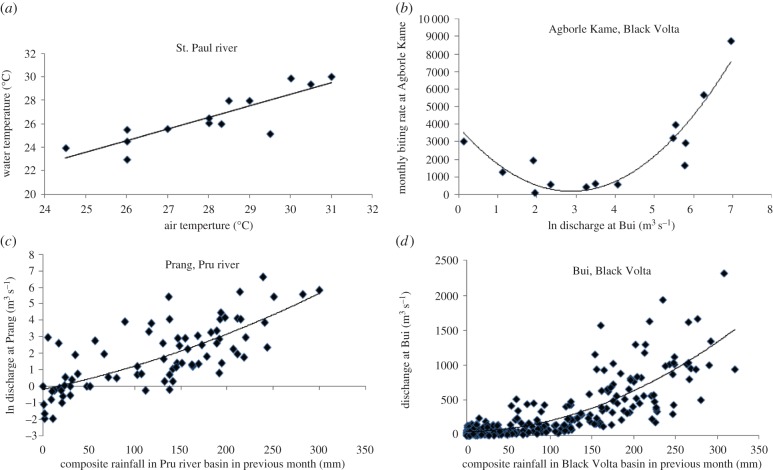
(*a*) The relationship between water temperature (*T*_w_, °C) and air temperature (*T*, °C) in the St. Paul river, Liberia. Data collected at the same times on each of several dates in 1968–1970 and 1989. The fitted equation is *T*_w_ = 0.9844*T*_w_ − 1.0352 (*R*^2^ = 0.7385). (*b*) The relationship between MBR of *S. damnosum* s.l. and average monthly ln (discharge in m^3^ s^−1^) of the Black Volta river at Agborle Kame, Ghana. Data for August and October to December 1974 and January to October 1975 inclusive. The fitted equation is MBR = 445.22(ln(discharge))^2^−2558.1ln(discharge) + 3843.7 (*R*² = 0.8587). (*c*) The relationship between average monthly ln (discharge in m^3^ s^−1^) of the Pru river and rainfall in the previous month (RF, mm); data from June 1957 to August 1967 inclusive. The fitted equation is ln(discharge) = 3E−05RF^2^ + 0.0117RF − 0.1953 (*R*^2^ = 0.5438). (*d*) The relationship between average monthly ln(discharge in m^3^ s^−1^) of the Black Volta river at Bui and rainfall in the previous month (mm); data for March 1951 to November 1975 inclusive. The fitted equation is ln(discharge) = 0.0135RF^2^ + 0.2384RF + 50.767 (*R*^2^ = 0.6675). (Online version in colour.)

Studies of the effects of temperature on development times of immature stages of *S. damnosum* s.l. have been based on water temperatures (table 1 shows temperature tolerances of different members of the *S. damnosum* complex in West Africa [24,25]), but climate change projections provide information on air temperatures so a relationship between the two was needed for conversions. River surface water temperatures vary with ambient temperature, elevation, canopy cover and river width. For instance, temperatures vary from about 24°C to more than 30°C in the St. Paul river but are much more stable, varying only from 22.5°C to 24.5°C in small streams in the Bong Hills (see fig. 2b of [13]). Although different functions will be needed for different rivers, river water temperatures (*T*_w_) are generally linearly related to ambient air temperatures (*T*), as illustrated by data for the St. Paul river (figure 1*a*) which can be modelled by the equation: *T*_w_ = 0.9844 *T* − 1.0352 (*R*^2^ = 0.74).

**Table 1.** Water temperature (°C) ranges of rivers in which different members of the *Simulium damnosum* complex have been found breeding in West Africa during wet and dry seasons, from [24,25].

taxon	water temperature range (°C), wet season	water temperature range (°C), dry season	water temperature range (°C), overall
*S. squamosum*	23–26.5	22–29	22–29 (mean 25)
*S. yahense*	23–25	24–30	23–30 (mean 25)
*S. sanctipauli*	26.5–28	29–33	26.5–33 (mean 27)
*S. soubrense*	24.5–28	30–33	24.5–33
*S. damnosum* s.str.	25–27	27.5–33	25–33 (mean 27)
*S. sirbanum*	25–26.5	27–33	25–33 (mean 27)

## 3. Effects of temperature

### (a) The impact of temperature on development of *Simulium* spp.

**Figure 2.**
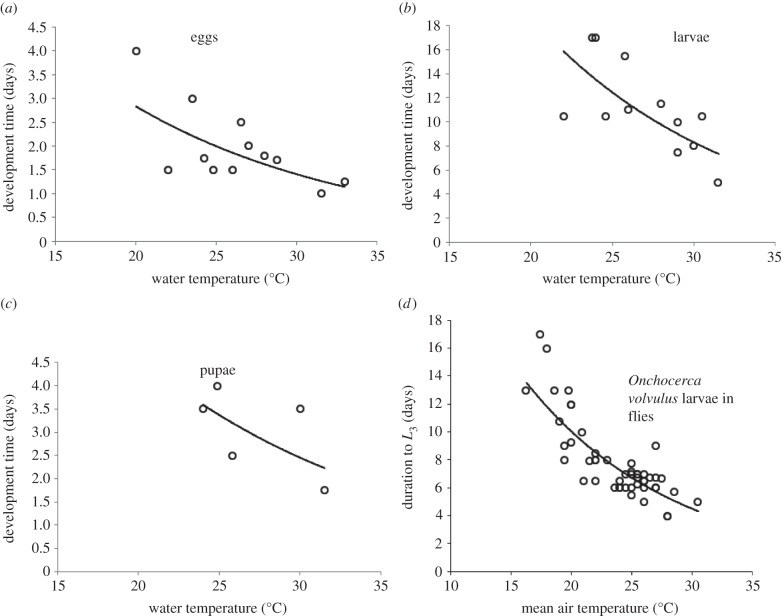
Development times of immature stages of *S. damnosum* s.l. at different temperatures. (*a*) Eggs, (*b*) larvae, (*c*) pupae (Adapted from [26,27]) and (*d*) the temperature-dependent development function for *O. volvulus* (data extracted from articles where experiments were conducted in a variety of onchocerciasis vectors *Simulium* spp., see the electronic supplementary material, S1). Fitted lines are exponential functions, for which the formulae for *a*–*c* are given in table 3 (*R*^2^ values = 0.473, 0.496 and 0.391 for *a*, *b* and *c*, respectively). The fitted line for D is Duration to *L*_3_ = 49.884e^−0.08*T*^ (where *T* = mean air temperature (°C); *R*^2^ = 0.695).

Cheke [26] collated data for the development of the different immature stages of the *S. damnosum* s.l. complex, a dataset which was extended by information from Crisp [27] and re-analysed. The temperature data for egg development ranged from 20°C to 33°C, those for larval development from 20°C to 31.5°C, and data for pupal development ranged from 24°C to 31.5°C; however, only five pupal data points were available. When temperature ranges were quoted in publications, weighted means were used in our analyses. All immature stages (eggs, larvae and pupae) showed a decrease in development time with increasing temperature, although data for low temperatures were unavailable. Functions of the form Δ_S_(*T*_w_) = *a* exp(−*bT*_w_) were fitted to the data, where Δ_S_(*T*_w_) is the development time of stage *s* (in days) as a function of water temperature *T*_w_ (°C), and *s* refers to eggs (*E*), larvae (*L*) or pupae (*P*). Parameter values for each stage are given in figure 2*a–c*.

### (b) The impact of temperature on development of *Onchocerca volvulus* within the simuliid vector

Although unlikely to affect the life history of adult worms in the homoeothermic human host, climate influences the development rate of the parasite within its vector. For instance, in *Simulium woodi* the worms take 16 days to reach the infective stage at 18°C but only 4 days at 28°C, the highest temperature at which the flies survived; at temperatures between 14°C and 17°C the larvae developed abnormally [28].

Data from 25 articles, describing 49 studies that gave information on both the development time of *O. volvulus* in a simuliid vector and the temperatures (16.3–30.5°C) at which the studies were carried out, were analysed. Mean temperatures were used when given, otherwise the midpoint of the provided range was taken as the mean. Times of first appearance of third stage (infective) larvae of *O. volvulus* (*L*_3_) were used whenever possible, but some studies used vague terminology such as ‘the development to abnormal sausage (pre-*L*_3_) stages took about 30 days' [28]. If a range was given, the shortest quoted time was used. The times for the first *L*_3_ appearances were chosen, because these were the most frequently quoted data and the most biologically meaningful. It also seemed more suitable for comparing data from such a wide variety of sources because selecting the midpoint of the range requires strong assumptions, particularly if development times are heterogeneous. It was assumed that although heavily infected flies may die quicker than flies with few larvae [29], larval development and survival times did not depend on how many microfilariae were ingested [30], so the microfilarial intake was not incorporated in the model. When second stage (*L*_2_) or *L*_3_ larvae were seen within the first 3 days, these larvae were ignored as being probably from a previous infection (as the fly-feeding experiments were rarely conducted with newly emerged flies, but mostly with wild caught host-seeking flies, which might have previously fed); microfilariae were grouped with the *L*_1_ stage when reported in the thorax. When the flies were checked every 24 h and the dead ones dissected to check for the presence of different larval stages [31–42], the developmental rates were estimated by maximum likelihood (using a multinomial distribution) [43] by fitting the following equations to data on larvae in each stage, *L*_1_(*t*), *L*_2_(*t*), *L*_3_(*t*), as a proportion of the total number of parasite larvae observed on each dissection day (*t*):3.1
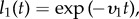
3.2
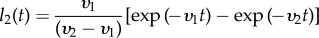
3.3
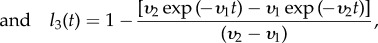
where *l*_1_, *l*_2_ and *l*_3_ are the proportions of larvae in each stage, and *υ*_1_ and *υ*_2_ are the rates of progression from *L*_1_ to *L*_2_, and from *L*_2_ to *L*_3_, respectively. A model without a delay between the first and the second stage larvae was tried initially, but including a delay gave a significantly better fit (according to the likelihood ratio test). The development rates were then converted into durations of development from *L*_1_ to *L*_2_, and from *L*_2_ to *L*_3_ by taking the reciprocal of the respective rates (table 2), and these values summed to obtain the overall duration from *L*_1_ to *L*_3_.

**Table 2.** Parameter values for rates of progression between *Onchocerca volvulus* first and second stage larvae (*υ*_1_), and between second and third (infective) stage larvae (*υ*_2_) in simuliid vectors. EIP: extrinsic incubation period (development time between microfilaria and infective larva). Parameters estimated by maximum likelihood.

species and locality	temperature (°C) (range)	delay as *L*_1_ (days)	*υ*_1_ (d^–1^)	*υ*_2_ (d^–1^)	EIP (days)	references
*Simulium guianense* s.l. S. Venezuela	20.0 (16.0–24.0)	5.56	1.413	0.331	9.28	[33]
*Simulium damnosum* s.l. Cameroon	21.5 (19.5–23.5)	4.37	0.552	0.569	7.94	[37,38]
*Simulium ochraceum* s.l. Guatemala	25.0	4.54	0.771	0.525	7.75	[31]
*Simulium guianense* s.l. S. Venezuela	25.0 (22.0–28.0)	3.32	0.455	0.610	7.16	[36]
*Simulium damnosum* s.l. Nigeria	27.0 (26.0–28.0)	4.03	—	—	5.09	[32]
*Simulium exiguum* s.l. Ecuador	27.5 (25.0–30.0)	2.53	0.449	0.518	6.69	[35]
*Simulium oyapockense* s.l./ *S. incrustatum*	28.5 (27.0–30.0)	3.77	0.742	1.565	5.76	[42]

Generally, the model of equations (3.2) and (3.3) gave good fits except when the transition from one larval stage to the next was highly synchronous. Eichner [37] quoted first appearance of *L*_3_ at 6 days post infection and all development complete by 9 days; the maximum likelihood estimation (MLE) gave an estimate of 7.9 days. Grillet *et al.* [42] quoted first appearance at 5 days and the MLE predicted 5.8 days. Matsuo *et al.* [31] did not check the flies on day 4 post infection, so it was assumed that the numbers and type of larvae observed on that day were the same as on day 3; the MLE fit estimated first appearance at 7.7 days and the first observation was at 8 days. Takaoka *et al*. [33] quoted seeing the first *L*_3_ on day 9 and the MLE fit predicted 9.3 days, but there were only 34 flies in their study. Basáñez *et al.* [36] first observed *L*_3_ in *S. guianense* at day 6, for which the MLE fit estimated 7.2 days. Not all published studies contained data suitable for estimation of *υ*_1_ and *υ*_2_ separately.

The development rates of *O. volvulus* for all simuliid species (collated in electronic supplementary material, S1) were used to obtain a temperature-dependent larval development function of the form Δ_L1+2_(*T*) = *a* exp(−*bT*), where Δ_L1+2_(*T*) is the duration in days of parasite larval development from *L*_1_ to *L*_3_ within simuliid vectors as a function of temperature (figure 2*d*). At temperatures above approximately 25°C, development can take less than 7 days and so, assuming a gonotrophic cycle of 3.5 days and that an infection was picked up at the first bite, a fly could transmit infective larvae as soon as after its second gonotrophic cycle.

### (c) The impacts of temperature and rainfall on blackfly populations and projections based on regressions of the mortality of the simuliid vector

Monthly data on mean numbers of flies caught per person, mean percentages of flies classified as parous, maximum, minimum and mean temperatures (°C), rainfall (mm) and river height (m, as measured at gauges in the St. Paul river) were available for Haindi and Gengema (where the vector is *S. soubrense*) and similar data but with river discharges (m^3^ s^−1^) instead of water levels were obtained for Agborle Kame and Asubende (where the vectors are the savannah species *S. damnosum* s.str. and *S. sirbanum*). To convert the Liberian water level data into approximate discharges, a relation linking the two for the Amou river in Togo was used, for which: ln(discharge) = 3.3 river height (m) − 1.29 (*P* < 0.0001; OCP data).

Figure 1*b* shows that fly numbers at Agborle Kame are dependent on river discharges, with higher numbers at low or high discharges than at intermediate levels. However, temperature is also an important variable as analysis of variance of the Liberian and Ghanaian data combined revealed that effects on monthly biting rates (MBR) of both average temperature (*T*_av_) and ln(discharge) were significant (*F* = 4.99, *P* < 0.03 and *F* = 5.66, *P* < 0.02, respectively, d.f. = 1/75). Since there was also a significant effect according to forest or savannah vectors (*F* = 91.46, *P* ≪ 0.0001), the following equations were derived: for the Liberian sites, ln(MBR) = 20.54–0.438 *T*_av_ − 0.0466 ln(discharge) (*P* < 0.002); and for the Ghanaian sites, ln(MBR) = 12.32–0.197 *T*_av_ + 0.152 ln(discharge) (*P* < 0.006). Analysis of data on the percentages of parous flies revealed a significant effect of vector species (*F* = 204.96, d.f. 1/75, *P* ≪ 0.0001), minimum temperature (*T*_min_, *F* = 13.06, *P* = 0.0005) and ln(discharge) (*F* = 5.90, *P* < 0.02), yielding the following equations: for the Liberian sites, asin(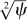) = 0.03995 − 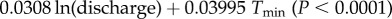, where *ψ* is the proportion of parous flies, and for the Ghanaian sites, asin(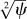) = 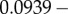 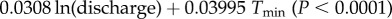.

Using the average monthly temperature (26.5°C) and the average estimated monthly discharge (214.25 m^3^ s^−1^) for the period 1973–1975 for Walker Bridge (7°33′ N, 9°53′ W), upstream of Haindi (River Discharge Database at http://www.sage.wisc.edu/riverdata/scripts/station_table.php?qual=32&filenum=2221), the above relation for Liberia predicts an MBR of 5901 flies per person. Assuming a forecast of a 1.2°C increase in temperature and a 25% reduction in river discharges, then only 3536 biting flies per person per month would be expected. Similarly for Ghana, using the mean monthly temperature (26.8°C) and the mean estimated discharge (128.3 m^3^ s^−1^) for Agborle Kame in 1975, the above relationship predicts an MBR of 2412 flies per person. Assuming a forecast of a 1.1°C increase in temperature and a 0.8% increase in river discharge, then only 1849 flies per person per month would be expected. A similar exercise for Asubende, using the mean temperature for 1979 (27.9°C) and mean monthly discharges for 1976 (494 m^3^ s^−1^) provides an estimate of 2206 bites per person per month. Assuming a temperature increase of 0.7°C and a 1.8% increase in river discharge this would reduce to 1865 bites per person per month. Such predictions could be improved by direct links to rainfall, commonly considered in the output of climate change models, since in both the Pru and Black Volta rivers discharges are related to their basins' composite rainfall in the previous month (figure 1*c,d*). However, the above extrapolations conflict with results from the dynamic model described in §4(a) and by calculations using matrix population models (RA Cheke 2012, unpublished data), possibly because they do not take into account the feedback loops and regulatory processes incorporated in the population dynamics model (i.e. essentially nonlinear processes); instead, they are based on linearized statistical relationships that do not account for the underlying biology. However, as will be seen in §4(a), the model includes temperature dependency of development and survival rates but does not yet include the effect of rainfall, warranting further work.

### (d) The impacts of temperature and rainfall on the mortality of the simuliid vector

Le Berre [44] pointed out that there are three main types of relationships between rivers and blackfly numbers: (i) those in which the flies' numbers are synchronous with river levels; (ii) those in which they vary inversely with river levels and (iii) those in which they are bimodal, such as at Agborle Kame, with peaks related to the availability of larval supports, such as vegetation, being optimal at low water levels and then again at high levels. Given this complexity it is difficult to generalize about effects of rainfall on fly survival or, indeed, on determinants of carrying capacities, so for this paper henceforth we restrict treatment of environmental influences on fly mortalities to temperatures. Furthermore, although there is some agreement that temperatures will increase, climate change projections for precipitation changes in West Africa vary according to the model used, although the likelihood of an increase in extreme events is generally accepted.

**Figure 3.**
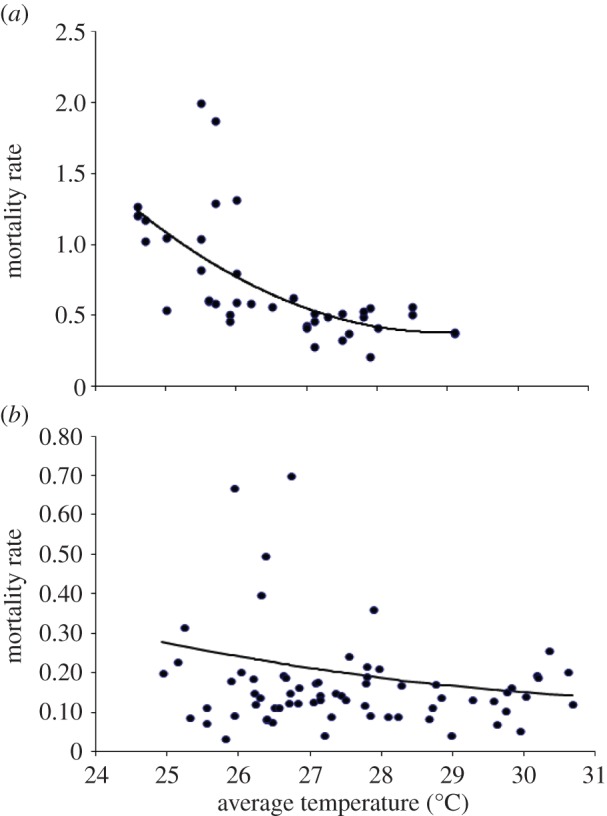
Mortality rates (*μ*_V_) of (*a*) *S. soubrense* at Gengema and Haindi, Liberia, in relation to average monthly temperature (°C) and (*b*) for *S. damnosum* s.str./*S. sirbanum* at Agborle Kame and Asubende. For means of calculation of the mortality rates from parous rates see text. The equation for the fitted line for (*a*) is *μ*_V_ = 0.0462*T*_av_^2^ – 2.671*T*_av_ + 38.988 (*R*^2^ = 0.426) and for (*b*) is *μ*_V_ = 0.0027 *T*_av_^2^ − 0.163 *T*_av_ + 2.602 (*R*^2^ = 0.031). Data from [13]. (Online version in colour.)

The proportion of parous flies (*ψ*) was used to calculate the probability of daily survival, *p*, by applying the parous rate formula [45] 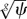, where *g* is the average duration between two consecutive blood meals (i.e. assuming gonotrophic concordance, the mean duration of the gonotrophic cycle), and the daily mortality rate, *μ*_V_, by taking –ln(*p*) under the assumption of exponential distribution of survival times and no difference in survival between nulliparous and parous flies. These mortality rates were calculated for the Haindi and Gengema data combined, as well as for the Agborle Kame and Asubende data combined, assuming *g* = 3.5 days [44,46], and were fitted by least-squares estimation to the monthly average temperature (*T*_mav_) with a polynomial of the form *aT*_mav_^2^ + *bT*_mav_ + *c*. Since the parous rates of *S. soubrense* in Liberia were very low (averaging 15%), the resulting mean life expectancy of the flies was approximately 2 days, possibly highlighting deficiencies with this method. By contrast, the mean parous rate of *S. damnosum* s.str./*S. sirbanum* in Ghana was 64%, and the mean life expectancy was 10 days (of the same order of magnitude as that estimated with laboratory data [29]). The parameter values for the flies in Liberia were *a* = 0.046; *b* = −2.671 and *c* = 38.99 (figure 3*a*); the values for the flies in Ghana were *a* = 0.003; *b* = −0.163 and *c* = 2.602 (figure 3*b*).

## 4. A mathematical model of *Simulium damnosum* s.l. population dynamics

### (a) The model

The following system of ordinary differential equations (ODEs; modified from [47]), describes the rates of change with respect to time (and dependent on temperature) of simuliid eggs *E*(*t*, *T*), larvae *L*(*t*, *T*), pupae *P*(*t*, *T*) and adult females distinguished between nulliparous *N*(*t*, *T*) and parous *Ψ*(*t*, *T*) flies. The total vector population, *V*(*t*, *T*) = *N*(*t*, *T*) + *Ψ*(*t*, *T*).4.1
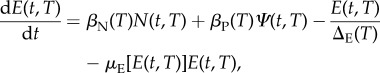
4.2
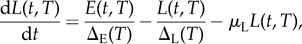
4.3
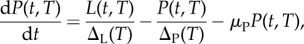
4.4
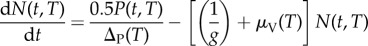
4.5
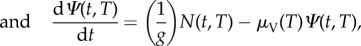
where *β*_N_(*T*) and *β*_P_(*T*) are the per nulliparous and per parous fly daily rates of oviposition (of viable eggs), respectively, which depend on temperature as described in §3(a); 1/Δ_E_(*T*) is the rate of progression from eggs to larvae (the reciprocal of the duration in the egg stage, dependent on temperature with a negative exponential relationship as described above); *μ*_E_[*E*(*T*, *t*)] is the *per capita* mortality rate of eggs (dependent on egg density according to Kyorku & Raybould [48]; *μ*_L_ and *μ*_P_ are the *per capita* mortality rates of larvae and pupae, respectively; 1/Δ_L_(*T*) is the rate of progression from larvae to pupae; 1/Δ_p_(*T*) is the rate of eclosion from pupae of adult (nulliparous) flies, half of which are females; *μ*_V_(*T*) is the *per capita* mortality rate of flies (which depends on temperature as described in §3d), and *g* is the mean duration of the gonotrophic cycle (as in §3d), which according to Takaoka *et al*. [11] decreases (by up to 2 days) with increasing temperature but does not vary substantially between 18°C and 28°C.

In blackflies, the adult host-seeking female population *V*(*t*,*T*) is often classified as nulliparous (those emerging from pupae and coming to bite for the first time), and parous (those which have fed before, laid eggs and are coming to bite for the second or third time, etc.), but unlike mosquitoes, it is not possible to distinguish between 1-parous, 2-parous, 3-parous, etc. The value of *g* is usually considered as being between 3 and 4 days [44,46], but may be as low as 2 or 2.5 days [11,49,50]. Following [47], the daily oviposition rates, *β*_N_(*T*) and *β*_P_(*T*), are estimated as the expected number of eggs oviposited in a fly's lifetime divided by the life expectancy. For nulliparous flies, the latter is the reciprocal of the rate at which they progress to the parous compartment (i.e. the duration of the gonotrophic cycle), and for parous flies it is the reciprocal of the parous mortality rate under the assumption of an exponential distribution of survival times. Therefore,4.6
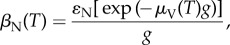
4.7
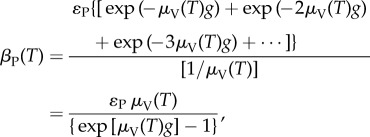
where *ɛ*_N_ and *ɛ*_P_ are the average number of (viable) eggs per oviposition laid by nulliparous and parous flies, respectively.

The expression exp(−*μ*_V_(*T*)*g*) + exp(−2*μ*_V_(*T*)*g*) + exp(−3*μ*_V_(*T*)*g*)+⋯ is the proportion of parous flies surviving consecutive gonotrophic cycles with mortality rate *μ*_V_(*T*). Nulliparous and parous flies have different fecundity rates (see §4b).

Values used for the parameters are explained in §4(b,c) and given in table 3. The model and the framework described below (§5) were coded in Berkeley Madonna v. 8.0.1 [55] using Runge–Kutta 4.

**Table 3.** Parameter definitions and values for blackfly population dynamics model. Note expressions for durations of immature stages account for conversion of air temperature to water temperature.

symbol	description	value	references
*E*(*t*, *T*)	no. eggs at time *t* and temperature *T*		
*L*(*t*, *T*)	no. larvae at time *t* and temperature *T*		
*P*(*t*, *T*)	no. pupae at time *t* and temperature *T*		
*V*(*t*, *T*)	no. vectors at time *t* and temperature *T*	*V* (*t*, *T*) = *N*(*t*, *T*) + *Ψ* (*t*, *T*)	
*N* (*t, T*)	no. nulliparous flies at time *t* and temperature *T*		
*Ψ* (*t*, *T*)	no. parous flies at time *t* and temperature *T*	*Ψ* (*t*, *T*) = *V*(*t*, *T*)/[exp(*μ*_V_*g*)−1]	
*β*_N_(*T*)	per nulliparous fly rate of oviposition at temperature *T*	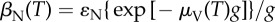	
*β*_P_(*T*)*ɛ*_N_	per parous fly rate of oviposition	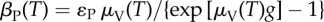	
*ɛ*_N_	no. eggs per nulliparous fly	432 eggs for *S. damnosum* s.str./*S. sirbanum*; 492 eggs for *S. squamosum*	[51,52]
*ɛ*_P_	no. eggs per parous fly	142 eggs for *S. damnosum* s.str./*S. sirbanum*; 215 eggs for *S. squamosum*	[51,52]
Δ_E_(*T*_W_)	duration of egg stage as a function of water temperature *T*_W_	11.493 exp(−0.0701*T*_W_)	this paper, figure 2*a*
Δ_L_(*T*_W_)	duration of larval stage as a function of water temperature *T*_W_	87.527 exp(−0.0785*T*_W_)	this paper, figure 2*b*
Δ_P_(*T*_W_)	duration of pupal stage as a function of water temperature *T*_W_	20.098 exp(−0.0699*T*_W_)	this paper, figure 2*c*
*T*_W_(*T*)	water temperature as a function of air temperature *T*	*T*_W_ = 0.9844 *T*–1.0352	this paper, figure 1*a*
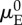	*per capita* background mortality rate of eggs	0.05 d^−1^	this paper
*α*_E_	density-dependent mortality rate of eggs	1.877 × 10^−6^ d^−1^ egg^−1^ (*S. soubrense*, Liberia)	this paper
		1.519 × 10^−5^ d^−1^ egg^−1^ (*S. damnosum* s.str./*S. sirbanum*, Ghana)	
*μ*_L_	*per capita* mortality rate of larvae	0.3 d^−1^	[53]
*μ*_P_	*per capita* mortality rate of pupae	0.1 d^−1^	[54]
*μ*_V_(*T*)	*per capita* mortality rate of vectors at temperature *T*	0.0462 *T*^2^ − 2.671 *T* + 38.99 d^−1^ (*S. soubrense*, Liberia)	this paper
		0.0027 *T*^2^ − 0.163 *T* + 2.602 d^−1^ (*S. damnosum* s.str./ *S. sirbanum*, Ghana)	
*Ψ*	proportion of parous flies	1/[exp(*μ*_V_*g*−1)]	this paper
*g*	length of gonotrophic cycle	3.5 days	[44,46]

### (b) Model parametrization: *fly fecundity*

The fecundity of nulliparous flies is greater than that of parous flies and both vary with fly size [51]. The average sizes of different cytoforms at a given time and place also vary depending on rainfall [56] via its effect on river discharges [57]. Thus, Cheke & Garms [52] derived the following expressions for the fecundity (eggs laid per oviposition) for nulliparous *S. damnosum* s.str./*S. sirbanum*: no. of oocytes (*O*) = 1370.79 thorax length (*λ*) in mm − 939.06, and for parous *S. damnosum* s.str.*/S. sirbanum*: *O*(*λ*) = 1063.95*λ* − 922.06; for nulliparous *S. squamosum*: *O*(*λ*) = 1230.65*λ* − 738.69, and for parous *S. squamosum*: *O*(*λ*) = 1081.33*λ* − 866.77. So, a nulliparous *S. squamosum* with a thorax length of 1.0 mm would be expected to lay *ɛ*_N_ = 492 eggs and a similar-sized *S. damnosum* s.str./*S. sirbanum* nulliparous fly would lay 432 eggs, with parous flies of similar sizes laying *ɛ*_P_ = 215 and 142 eggs, respectively. Data on fecundities of members of the *S. sanctipauli* sub-complex are lacking but their adults are known to be larger than *S. damnosum* s.str./*S. sirbanum* when they are sympatric [56] so we used the *S. squamosum* relations for *S. soubrense* in Liberia. For this paper, the effect of intraspecific fly size variability is ignored.

### (c) Model parametrization: *mortality rates*

#### (i) Eggs

Elsen [58] reported that only 2.7% of eggs reached the pupal stage, which for a development period from egg to pupa of roughly 14 days, yields a daily mortality rate of 0.77; in the absence of reliable field data for egg survival, it is therefore assumed that the *per capita* mortality rate of eggs is *μ*_E_ = 0.8 d^−1^. However, given that this was measured in the field, the value corresponds to overall mortality and not to background mortality. In addition, it has been reported that egg mortality increases with egg mass density [48], but the functional form of that relationship is unknown. Following [59], a linear function was chosen, so that 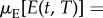 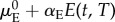 with 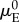 the background rate of egg mortality and *α*_E_ = the rate of excess mortality per additional egg in the egg mass. Following [47], and to stabilize the population, the mortality rate of eggs was parametrized in terms of a carrying capacity (*K*) of adult vectors. Therefore, the differential equation for the simuliid eggs can be re-written as follows:4.8
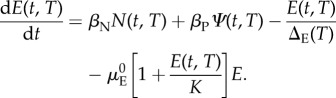


So that 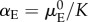. The value of 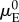 was taken as 0.05 d^−1^.

To derive an expression for *K*, the equations for the blackfly population dynamics were set to zero and equilibrium expressions for each stage were obtained (omitting temperature dependence for simplicity; see electronic supplementary material, S2, where the formula for the basic reproduction number or ratio, *R*_0_, of the blackfly populations, 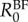, based on equations (4.2)–(4.5) and (4.8) is also given; if 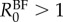, the unique positive equilibrium of the model is globally asymptotically stable (electronic supplementary material, S3),4.9
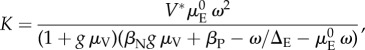
where *ω* = 2Δ_E_(1 + *μ*_L_Δ_L_)(1 + *μ*_P_Δ_P_) (1 + *μ*_V_*g*) *μ*_V_.

In order to obtain values for *K* from equation (4.9), it was necessary to estimate the equilibrium vector population, V*, which was calculated for the Liberia and Ghana data on pre-control daily vector biting rates (DBR*), human population size (*H*), length of gonotrophic cycle (*g*) and proportion of blood meals taken on humans (*h*), following 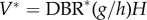 [12]. For *S. soubrense* in Liberia *h* = 0.5 based on zoophagy data [13,15] and *H* = 100 in Gengema [60]. For *S. damnosum* s.str./*S. sirbanum* in Ghana, *h* = 0.74 (authors' unpublished data on blood-meal analyses providing proportions of flies biting cattle, sheep, goats, pigs, dogs and unknown non-human hosts) and *H* = 462 (the average of the human population sizes in Asubende and Agborle Kame assessed by Ghana Health Services in 1988).

#### (ii) Larvae

Although no data on larval mortality were available, the *per capita* larval mortality was assumed to be *μ*_L_ = 0.3 d^−1^ since Davies *et al*. [53] found that a survival rate of 0.7387 per day was needed to stabilize their model that used field estimates for most of its other parameters.

#### (iii) Pupae

Edwards & Trenholme [54] reported a proportion of pupal survival of 0.75 over a 3-day experiment, equivalent to 0.91 per day, so a *per capita* pupal mortality rate of *μ*_P_ = 0.1 d^−1^ was assumed.

#### (iv) Adults

Fly mortality rates were estimated as described in §3(d) and followed a parabolic functional form with temperature (figure 3).

### (d) Model simulations of *Simulium soubrense* population dynamics in Liberia

**Figure 4.**
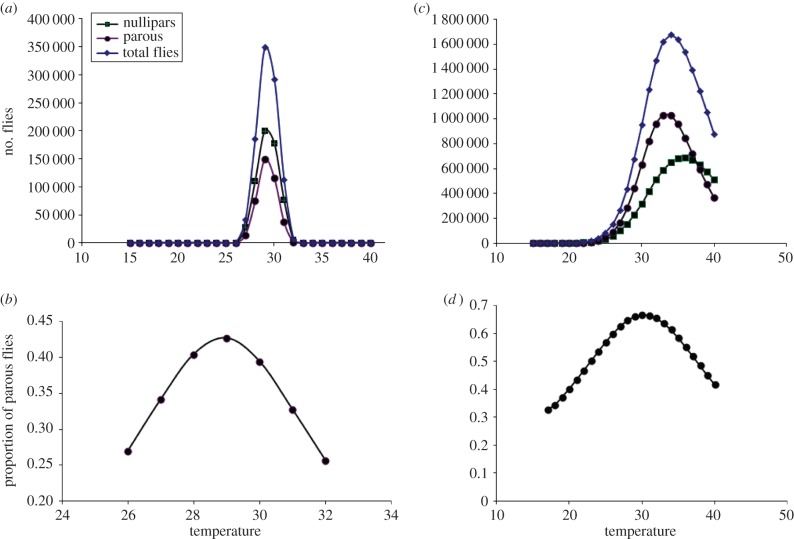
Output of blackfly population models with parameters for Liberia (forest) (*a*,*b*) and Ghana (savannah) (*c*,*d*). (*a,c*) Equilibrium densities of total numbers of flies (diamonds), nulliparous flies (squares) and parous flies (circles) at different temperatures (°C). (*b,d*) Proportions of parous flies at different temperatures (°C). (Online version in colour.)

The model was run until equilibrium values were obtained for the state variables for varying temperatures using values for *K* derived from mean biting rates at Haindi and Gengema for the air temperature range of 25–29°C, and calculating pre-imaginal development rates according to water temperatures derived from the regression between air and water temperatures. The model predicted total numbers of biting flies, nulliparous flies and parous flies as depicted in figure 4*a*. This parametrization of the model only gives positive results between 26°C and 32°C and indicates that *S. soubrense* populations would peak at 29°C before declining. Over this temperature range, the model predicts parous rates to vary as shown in figure 4*b*. With *T* = 30, 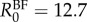.

### (e) Model simulations of *Simulium damnosum* s.str.*/Simulium sirbanum* population dynamics in Ghana

At equilibrium for a range of temperatures, using values for *K* derived from average biting rates at Agborle Kame and Asubende for the air temperature range of 25–31°C, and calculating pre-imaginal development rates according to water temperatures as described in §3(a), with parameter values as in table 3 and adult mortalities as described for *S. damnosum* s.str.*/S. sirbanum* in §3d, the model predicts total numbers of biting flies, nulliparous flies and parous flies as depicted in figure 4*c*. This parametrization of the model gives positive results above 18°C and shows that populations will peak at 34°C before declining. No simulations were run at temperatures of more than 40°C. The model's parous rate predictions are shown in figure 4*d*. With *T* = 30, 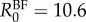.

## 5. A mathematical model of the transmission dynamics of *Onchocerca volvulus*

In the light of the above, it is possible to modify the model presented in [12] to account for the population dynamics of vectors and the effects of temperature on simuliids and on *O. volvulus* within the simuliids. The equations for the modified model are as follows:5.1
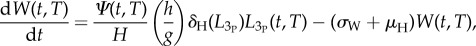
5.2
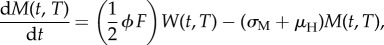
5.3
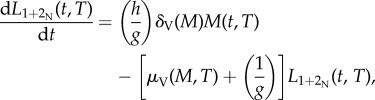
5.4
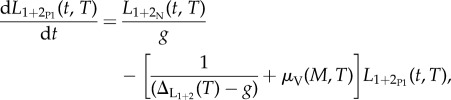
5.5
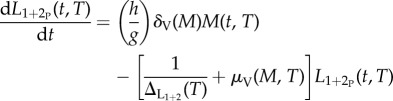
5.6
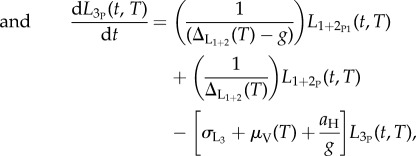
where *W* and *M* denote the mean number of adult worms per host and of microfilariae per milligram of skin, respectively; *L*_1+2_N__ and *L*_1+2_P__ are the mean numbers of parasite larvae developing in the thoracic muscles of nulliparous and parous flies, respectively (with *L*_1+2_P1__ being larvae in flies in their first parous cycle), 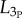 is the mean number of infective larvae in parous flies; *H* is the human population density; *h* is the proportion of blood meals taken on humans; 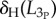 is the probability of parasite establishment within the human host, dependent on the intensity of exposure to infective larvae, with equation 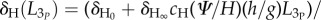 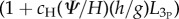, omitting the time and temperature dependencies to facilitate notation; *δ*_V_(*M*) is the probability of parasite establishment within the vector host, dependent on the intensity of microfilarial infection in the skin, with equation 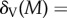 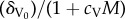, again omitting time and temperature dependencies, with 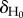 and 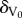 representing the maximum probability of parasite establishment within humans and vectors that applies when the intensity of exposure to infective larvae or the intensity of microfilarial infection tend to zero; 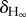 is the probability of parasite establishment within humans when the intensity of exposure to infective larvae is very large (with 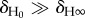, the latter can be set equal to zero), and *c*_H_ and *c*_V_ represent the magnitude of (negative) density dependence operating upon parasite establishment within humans and vectors, respectively (the latter can be set equal to zero for forest flies [43]); *σ*_W_, *σ*_M_ and 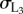 are the *per capita* mortality rates of adult worms, microfilariae and infective larvae (ignoring the mortalities of *L*_1_ and *L*_2_ larvae as it was not possible to estimate these from the data, and thus it is assumed that once in the thorax, parasite larvae will progress to the *L*_3_ stage); *μ*_H_ is the mortality rate of humans; *μ*_V_ is the mortality rate of vectors, which in addition to being temperature dependent, is also dependent on the microfilarial load ingested by the flies [29], with functional form 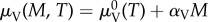, where 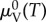 is the background rate of mortality dependent on temperature as described in §3(d), and *α*_V_ is the per ingested microfilaria excess mortality rate of the vectors (assuming that this rate is independent of temperature); finally, *a*_H_ is the proportion of infective larvae shed per bite. Parameter values used are the averages given in table 2 of Basáñez & Boussinesq [12].

**Figure 5.**
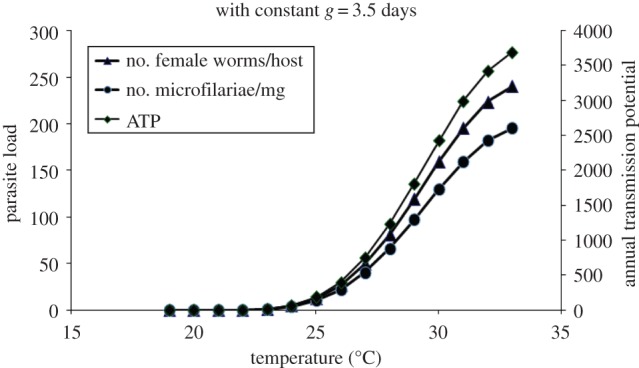
Output of combination of blackfly population model with parameters for Ghana (savannah) and model for onchocerciasis in the human host showing variation of equilibrium values of numbers of female worms per host (triangles), number of microfilariae per milligram of skin (circles) and ATP of the flies (diamonds) with temperature. (Online version in colour.)

Results of running the model with *S. damnosum* s.str./*S. sirbanum* (Ghana data) parameters for 100 years to ensure endemic equilibrium at air temperatures from 19°C to 33°C are shown in figure 5. The annual transmission potential (ATP, i.e. the number of infective larvae potentially received annually per person [61]), numbers of female worms per human host and numbers of microfilariae per milligram in the skin of human hosts all gradually rise with temperature, but the rate of increase begins to decelerate above 30°C.

## 6. Discussion

The extrapolations based on regressions of field data on blackfly numbers with temperatures and river discharges suggested that fly populations are likely to decrease with increasing temperatures and decreasing, or slightly increasing, river discharges, with the magnitudes of the assumed environmental changes based on regional climate change model scenarios. By contrast, the output of the ODE model, albeit lacking explicit inputs regarding precipitation or river discharges, predicted that fly numbers would have a humped distribution peaking at average air temperatures of 29°C in Liberia and 34°C in Ghana. Given that recent average monthly air temperatures in the study sites were 26.5°C and 26.8°C in Liberia and Ghana, respectively, and are expected to increase by up to 1.1°C or 1.3°C on the basis of conservative climate change scenarios, both countries are likely to see substantial increases in numbers of onchocerciasis vectors in the next few decades on the basis of the ODE model, because of accelerating rates of blackfly development with increasing temperature. However, although there is more uncertainty regarding future changes in precipitation, climate change models for Liberia suggest that the discharge of the St. Paul river will decrease by 0.7–25%, but in Ghana increases of 0.8% in the Black Volta river and 1.8% in the Pru river are expected. Depending on how such changes affect fly numbers it is possible, if river topographies allow lower discharges to lead to fewer flies, that in forested areas of West Africa such as Liberia onchocerciasis transmission may decrease (as implied by the relations based on empirical data) but in savannah zones such as northern Ghana it might increase. The latter conclusion is supported by the results obtained when the vector model was linked to the dynamics of the parasite in humans and vectors which indicated increases in worm burden with increasing temperature in Ghana up to temperatures of 33°C, because of accelerating development of parasite larvae within vectors. Reconciling the contrasting results between those from the field data statistical relationships and the ODE dynamics model is imperative and highlights the deficiencies of statistical (linearized) relationships as opposed to nonlinear dynamics on the one hand, and the need to refine the dynamic model by including dependencies of the vector carrying capacity with rainfall and river levels on the other hand. Also, the modelling of vector mortality was relatively simplistic, assuming an exponential distribution of survival times and a constant (and equal) mortality rate for nulliparous and parous flies. Further work is necessary to better understand the dependency of vector survival and increasing temperature, and laboratory data on neotropical vectors (not shown) suggest that vector mortality may increase with temperature at rates higher than those derived from the observed proportions of parous flies described here. Also, the statistical relationships were derived from existing temperature and river discharge ranges, whereas the dynamic projections predict peak fly numbers to occur above current maximal temperatures, so it is possible that the shape of the empirical functions will change as temperature and rainfall patterns become more extreme.

Although there have been previous studies modelling blackfly population dynamics most have been based on difference equations [53,59,62] and, so far as we are aware, the differential equation model presented here is the first of its kind. While it was designed to be as realistic as possible on the basis of current knowledge, it could be improved by incorporating the effects of rainfall, perhaps by linking precipitation to river discharges, to describe the carrying capacity of eggs explicitly, as opposed to the current construct derived from the mathematical characteristics of the functions used and based on crude estimates of host populations available to the flies, which are probably very low as they are based on sizes of villages where the flies were caught. In addition, more information is needed on the effects of environmental variables on fly mortalities. Indeed the differences in the dynamics of the Liberian and Ghanaian flies may be accounted for by the different mortality/temperature relations used. It is also possible that the gonotrophic cycle length of *S. damnosum* s.l. is either less than the 3.5 days assumed or that it is temperature dependent, as is the case with other species [11], which will be analysed in future modelling work. Also, the relations for development times of the immature stages of the flies are based on few data and only for water temperatures ranging from 20°C to 33°C, so caution is needed for any forecasts of fly numbers above 33°C. Furthermore as with most, but not all, population dynamics models, immigration and emigration are ignored, even though it is known that savannah members of the *S. damnosum* complex may migrate up to 300 km [63].

Irrespective of how *S. damnosum* s.l. population densities will alter with climate change, it is likely that increasing temperatures will lead to changes in the geographical distribution of some species. For instance, *S. squamosum* and *S. yahense* are adapted to colder water temperatures than *S. damnosum* s.str. and *S. sirbanum* (table 1) so the latter may replace the former at some sites. Such replacements of forest species by savannah species have already occurred in response to habitat changes and are likely to lead to deteriorating epidemiological outcomes [64,65]. Other ecological changes may also have already occurred, perhaps in response to climatic changes during the past 40 or more years. However, even if we had had access to a complete 1974–2001 dataset for the OCP area, any trends would have been difficult to discern there, as they are likely to have been masked by OCP's vector control activities.

The research presented here is timely, as in 2012 the Disease Reference Group for Helminth Infections (DRG4) of the UNICEF/UNDP/World Bank/WHO Special Programme for Research and Training in Tropical Diseases (TDR) identified a need to develop models to investigate the effects of climate change on helminthiases and their control. They recommended conducting literature reviews, experimental/observational studies and parameter estimation to calibrate models on the interaction between the biology of the infections and climate-driven environmental variables [66]. Among the helminth infections transmitted by haematophagous vectors was onchocerciasis, a neglected tropical disease (NTD) earmarked for elimination in the American continent by 2015 and in selected African countries by 2020 according to the World Health Organization (WHO) roadmap for accelerating progress to overcome the impact of NTDs [67]. As our results are inconclusive and conflicting, it is clear that further research is needed before the level of uncertainty surrounding how climate change will affect onchocerciasis transmission, and its control can be reduced. Such further work could encompass seasonality, fly migrations including those of savannah species into forest areas and *vice versa*, modelling of transmission in the forest (not covered here through lack of space), spatio-temporal changes in human and non-human blood host populations, effects of chemotherapeutic treatments, spatial variation in parous rates and climate-related variations in parameters such as gonotrophic cycle lengths.

## Supplementary Material

Supplement 1

## Supplementary Material

Supplement 2

## Supplementary Material

Supplement 3

## References

[1] Siraj AS Santos-Vega M Bouma MJ Yadeta D Ruiz Carrascal D Pascual M 2014 Altitudinal changes in malaria incidence in highlands of Ethiopia and Colombia. Science 343, 1154–1158. (10.1126/science.1244325) 24604201

[2] Bradley JE Whitworth J Basáñez MG 2005 Onchocerciasis. In Topley and Wilson‘s microbiology and microbial infections, volume Parasitology, (eds Cox FEG Wakelin D Gillespie SH Despommier DD), pp. 781–801, 10th edn London, UK: Hodder Arnold.

[3] Little MP Breitling LP Basáñez MG Alley ES Boatin BA 2004 Association between microfilarial load and excess mortality in onchocerciasis: an epidemiological study. Lancet 363, 1514–1521. (10.1016/S0140-6736(04)16151-5) 15135599

[4] Amazigo U Noma M Bump J Benton B Liese B Yaméogo L Zouré H Sékétéli A 2006 Onchocerciasis. In Disease and mortality in sub-Saharan Africa (eds Jamison DT Feachem RG Makgoba MW Bos ER Baingana FK Hofman KJ Rogo KO), pp. 215–222, 2nd edn Washington, DC: The World Bank (http://www.ncbi.nlm.nih.gov/books/NBK2287/)

[5] Basáñez MG Pion SDS Churcher TS Breitling LP Little MP Boussinesq M 2006 River blindness: a success story under threat? PLoS Med. 3, e371 (10.1371/journal.pmed.0030371) 17002504 PMC1576321

[6] Vajime CG Dunbar RW 1975 Chromosomal identification of eight species of the subgenus *Edwardsellum* near and including *Simulium* (*Edwardsellum*) *damnosum* Theobald (Diptera: Simuliidae). Tropenmed. Parasitol. 26, 111–138. 1145723

[7] Cheke RA Garms R 2013 Indices of onchocerciasis transmission by different members of the *Simulium damnosum* complex conflict with the paradigm of forest and savanna parasite strains. Acta Trop. 125, 42–53. (10.1016/j.actatropica.2012.09.002) 22995985

[8] Post RJ 2013 Stability and change in the distribution of cytospecies of the *Simulium damnosum* complex (Diptera: Simuliidae) in southern Ghana from 1971 to 2011. Parasit. Vectors 6, 205 (10.1186/1756-3305-6-205) 23849451 PMC3727979

[9] Ocran MH Davies JB Agoua H Gboho C Ouedraogo J 1982 Water temperatures in *S. damnosum* breeding rivers of the Onchocerciasis Control Programme area. Geneva, Switzerland: World Health Organization unpublished mimeograph report WHO/VBC/82.848.

[10] Philippon B 1977 Étude de la transmission d’*Onchocerca volvulus* (Leuckart, 1893) (Nematoda, Onchocercidae) par *Simulium damnosum* Theobald, 1903 (Diptera: Simuliidae) en Afrique tropicale. Trav. Doc. O.R.S.T.O.M. 63, 308.

[11] Takaoka H Ochoa JO Juarez EL Hansen KM 1982 Effects of temperature on development of *Onchocerca volvulus* in *Simulium ochraceum*, and longevity of the simuliid vector. J. Parasitol. 68, 478–483. (10.2307/3280961) 7097444

[12] Basáñez MG Boussinesq M 1999 Population biology of human onchocerciasis. Phil. Trans. R. Soc. Lond. B 354, 809–826. (10.1098/rstb.1999.0433) 10365406 PMC1692549

[13] Garms R 1973 Quantitative studies on the transmission of *Onchocerca volvulus* by *Simulium damnosum* in the Bong Range, Liberia. Z. Tropenmedizin Parasitol. 24, 358–372. 4744222

[14] Post RJ 1986 The cytotaxonomy of *Simulium sanctipauli* and *Simulium soubrense* (Diptera: Simuliidae). Genetica 69, 191–207. (10.1007/BF00133522)

[15] Garms R 1987 Infection rates and parasitic loads of *Onchocerca volvulus*, and other filariae, in *Simulium sanctipauli* s.l. and *S. yahense* in a rain-forest area of Liberia. Trop. Med. Parasit. 38, 201–204. 3432956

[16] FAO. 2006 New_LocClim, Local Climate Estimator Version 1.10. Rome, Italy: Environment and Natural Resources Service—Agrometeorology Group, FAO/SDRN (ftp://extftp.fao.org/SD/SDR/Agromet/New_LocClim/) (accessed 12 July 2011).

[17] Van der Linden P Mitchell JFB (eds). 2009 ENSEMBLES: *climate change and its impacts*. Summary of Research and Results from the ENSEMBLES Project Exeter, UK: Meteorological Office Hadley Centre.

[18] Paeth H 2001 Progress in regional downscaling of West African precipitation. Atmos. Sci. Lett. 12, 75–82. (10.1002/asl.306)

[19] Arnell NW 1998 Climate change and water resources in Britain. Clim. Change 39, 83–110. (10.1023/A:1005339412565)

[20] Gellens D Roulin E 1998 Stream flow response of Belgian catchments to IPCC climate change scenarios. J. Hydrol. 210, 242–258. (10.1016/S0022-1694(98)00192-9)

[21] Budyko MI 1974 Climate and life. New York, NY: Academic Press.

[22] WHO. 2002 Success in Africa; the onchocerciasis control programme in West Africa, 1974–2002. Geneva, Switzerland: World Health Organization.

[23] Middelkoop H 2001 Impact of climate change on hydrological regimes and water resources management in the Rhine Basin. Clim. Change 49, 105–128. (10.1023/A:1010784727448)

[24] Grunewald J 1976 The hydro-chemical and physical conditions of the environment of the immature stages of some species of the *Simulium* (*Edwardsellum*) *damnosum* complex (Diptera). Tropenmedizin Parasitol. 27, 438–454. 12601

[25] Quillévéré D 1979 Contribution à l’étude des caractéristiques taxonomiques, bioécologiques et vectrices des membres du complexe *Simulium damnosum* presents en Côte d'Ivoire. Paris: Travaux et Documents de l'ORSTOM.

[26] Cheke RA 2012 The thermal constant of the onchocerciasis vector *Simulium damnosum* s.l. in West Africa. Med. Vet. Entomol. 26, 236–238. (10.1111/j.1365-2915.2011.00980.x) 21988115

[27] Crisp HK 1956 Simulium and onchocerciasis in the northern territories of the Gold Coast. London, UK: HK Lewis and Co.

[28] Wegesa P 1966 Some factors influencing the transmission of *Onchocerca volvulus* by *Simulium woodi*. Ann. Rep. East Afr. Inst. Mal. Vect. Dis. 14–17.

[29] Basáñez MG Townson H Williams JR Frontado H Villamizar NJ Anderson RM 1996 Density-dependent processes in the transmission of human onchocerciasis: relationship between microfilarial intake and mortality of the simuliid vector. Parasitology 113, 331–355. (10.1017/S003118200006649X) 8873475

[30] Kershaw WE 1958 The population dynamics of infection with *Onchocerca volvulus* in the vector *Simulium damnosum**.* In Proc. 10th Int. Congr. Entomol., Montreal, Canada, *17–25 August 1956* (ed. E Becker), vol. 3, pp. 499–501. Ottawa, Canada: Mortimer.

[31] Matsuo K Okazawa T Onishi O Ochoa A 1980 Experimental observation of developmental period of *Onchocerca volvulus* in black fly, *Simulium ochraceum**.* Jap. J. Parasitol. 29, 13–17.

[32] Gemade EI Dipeolu OO 1983 Onchocerciasis in Benue State of Nigeria. A laboratory study of the development of *Onchocerca volvulus* in wild *Simulium damnosum* experimentally fed on an infected volunteer. Ann. Soc. Belg. Med. Trop. 63, 219–225. (http://lib.itg.be/open/asbmt/1983/1983asbm0219.pdf) 6660951

[33] Takaoka H Suzuki H Noda S Tada I Basáñez MG Yarzábal L 1984 Development of *Onchocerca volvulus* larvae in *Simulium pintoi* in the Amazonas region of Venezuela. Am. J. Trop. Med. Hyg. 33, 414–419. (http://hdl.handle.net/10069/21852) 6731673 10.4269/ajtmh.1984.33.414

[34] Shelley AJ Dias AP Moraes MA Procunier WS 1987 The status of *Simulium oyapockense* and *S. limbatum* as vectors of human onchocerciasis in Brazilian Amazonia. Med. Vet. Entomol. 1, 219–234. (10.1111/j.1365-2915.1987.tb00348.x) 2979535

[35] Takaoka H 1987 Studies on the role of three anthropophilic blackfly species as the vectors of human onchocerciasis in Ecuador. In A comparative study on onchocerciasis between South and Central Americas (ed. Tada I), pp. 69–71, Kumamoto, Japan: Shimoda.

[36] Basáñez MG Yarzábal L Takaoka H Suzuki H Noda S Tada I 1988 The vectoral role of several blackfly species (Diptera: Simuliidae) in relation to human onchocerciasis in the Sierra Parima and Upper Orinoco regions of Venezuela. Ann. Trop. Med. Parasitol. 82, 597–611. 3256278 10.1080/00034983.1988.11812296

[37] Eichner M 1989 *Onchocerca volvulus* (Nematoda, Filarioidea) und *Simulium damnosum*-Komplex (Diptera): Die Entwicklung intrathorakal injizierter Mikrofilarien in verschiedenen Überträgerspecies Kameruns. Diplomarbeit, Universität Tübingen, Germany: Fakultät für Biologie.

[38] Eichner M Renz A Wahl G Enyong P 1991 Development of *Onchocerca volvulus* microfilariae injected into *Simulium* species from Cameroon. Med. Vet. Entomol. 5, 293–297. (10.1111/j.1365-2915.1991.tb00555.x) 1768922

[39] Grillet ME 1993 Estudio de *Simulium metallicum*, vector principal de oncocercosis en el norte de Venezuela: ecologia, competencia vectorial y citotaxonoma. PhD thesis, Caracas, Venezuela: Universidad Central de Venezuela.

[40] Grillet ME Botto C Basáñez MG Barrera R 1994 Vector competence of *Simulium metallicum* s.l. (Diptera: Simuliidae) in two endemic areas of human onchocerciasis in northern Venezuela. Ann. Trop. Med. Parasitol. 88, 65–75. 8192518 10.1080/00034983.1994.11812837

[41] Wetten S Collins RC Vieira JC Marshall C Shelley AJ Basáñez MG 2007 Vector competence for *Onchocerca volvulus* in the *Simulium* (*Notolepria*) *exiguum* complex: cytoforms or density-dependence? Acta Trop. 103, 58–68. (10.1016/j.actatropica.2007.05.009) 17618859

[42] Grillet ME Villamizar NJ Frontado HL Cortez J Escalona M Botto C Basáñez MG 2008 Vector competence of *Simulium oyapockense* s.l. and *S. incrustatum* for *Onchocerca volvulus*: implications for ivermectin-based control in the Amazonian focus of human onchocerciasis, a multi-vector-host system. Acta Trop. 107, 80–89. (10.1016/j.actatropica.2008.04.021) 18538741

[43] Basáñez MG Churcher TS Grillet ME 2009 *Onchocerca-Simulium* interactions and the population and evolutionary biology of *Onchocerca volvulus*. Adv. Parasitol. 68, 263–313. (10.1016/S0065-308X(08)00611-8) 19289198

[44] Le Berre R 1966 Contribution à l’étude biologique et écologique de *Simulium damnosum* Theobald, 1903 (Diptera, Simuliidae). Mém. ORSTOM 17, 1–204.

[45] Davidson G 1954 Estimation of the survival rate of anopheline mosquitoes in nature. Nature 174, 792–793. (10.1038/174792a0) 13214009

[46] Millest AL Cheke RA Howe MA Lehane MJ Garms R 1992 Determining the ages of adult females of the *Simulium damnosum* complex (Diptera: Simuliidae) by the pteridine accumulation method. Bull. Entomol. Res. 82, 219–226. (10.1017/S0007485300051762)

[47] White MT Griffin JT Churcher TS Ferguson NM Basáñez MG Ghani AC 2011 Modelling the impact of vector control interventions on *Anopheles gambiae* population dynamics. Parasit. Vectors 4, 153 (10.1186/1756-3305-4-153) 21798055 PMC3158753

[48] Kyorku CA Raybould JN 1987 Preliminary studies on egg-mass development in the *Simulium damnosum* Theobald complex (Diptera: Simuliidae). Int. J. Trop. Insect Sci. 8, 311–316. (10.1017/S1742758400005294)

[49] Holmes PR Birley MH 1987 An improved method for survival rate analysis from time series of haematophagous dipteran populations. J. Anim. Ecol. 56, 427–440. (10.2307/5058)

[50] Cheke RA 1995 Cycles in daily catches of members of the *Simulium damnosum* species complex. Trop. Med. Parasitol. 46, 247–252. 8826105

[51] Cheke RA Garms R Kerner M 1982 The fecundity of *Simulium damnosum* s.l. in northern Togo and infections with *Onchocerca* spp. Ann. Trop. Med. Parasitol. 76, 561–568. 6891577 10.1080/00034983.1982.11687581

[52] Cheke RA Garms R 1986 Fecundities of different members of the *Simulium damnosum* species complex in Togo*.* Trans. R. Soc. Trop. Med. Hyg. 80, 489–490. (10.1016/0035-9203(86)90355-X) 3798548

[53] Davies JB Weidhaas DE Haile DG 1987 Models as aids to understanding onchocerciasis. In Black flies: ecology, population management and annotated world list (eds. Kim KC Merritt RW), pp. 396–407. University Park, PA: Pennsylvania State University.

[54] Edwards AJ Trenholme AAG 1976 Diel periodicity in the adult eclosion of the blackfly *Simulium damnosum* Theobald, in the Ivory Coast. Ecol. Entomol. 1, 279–282. (10.1111/j.1365-2311.1976.tb01233.x)

[55] Macey RI Oster GF 2000 Berkeley Madonna. Version 8.0.1 for Windows. 1442-A Walnut Street #392-GO, Berkeley, CA 94709-1405, USA.

[56] Cheke RA Harris JRW 1980 Seasonal size variation in females of the *Simulium damnosum* complex in the Ivory Coast. Tropenmed. Parasitol. 31, 381–385. 6255641

[57] Cheke RA Sowah SA Avissey HSK Fiasorgbor GK Garms R 1992 Seasonal variation in onchocerciasis transmission by *Simulium squamosum* at perennial breeding sites in Togo. Trans. R. Soc. Trop. Med. Hyg. 86, 67–71. (10.1016/0035-9203(92)90445-I) 1566312

[58] Elsen P 1987 Notes sur la dynamique des populations préimaginales de *Simulium damnosum* s.l. (Diptera: Simuliidae) de Côte d'Ivoire et du Burundi. Revue Zool. Afr. 101, 525–539.

[59] Cheke RA Asomaning ME 1995 A simulation model of onchocerciasis transmission by different members of the *Simulium damnosum* species complex, p. 168 In *Proc. European Conference on Tropical Medicine*, Hamburg, Germany, 22–26 October 1995, p. 168. Oxford, UK: Blackwell Science.

[60] Frentzel-Beyme RR 1973 The prevalence of onchocerciasis and blindness in the population of the Bong range, Liberia. Zeitschrift Tropenmedizin Parasitol. 24, 339–357. 4744221

[61] Walsh JF Davies JB Le Berre R Garms R 1978 Standardisation of criteria for assessing the effect of *Simulium* control in onchocerciasis control programmes. Trans. R. Soc. Trop. Med. Hyg. 72, 675–676. (10.1016/0035-9203(78)90039-1) 734734

[62] Birley MH Walsh JF Davies JB 1983 Development of a model for *Simulium damnosum* s.l. recolonization dynamics at a breeding site in the Onchocerciasis Control Programme area when control is interrupted. J. Appl.Ecol. 20, 507–519. (10.2307/2403523)

[63] Garms R Walsh JF Davies JB 1979 Studies on the reinvasion of the Onchocerciasis Control Programme in the Volta River basin by *Simulium damnosum* s.l. with emphasis on the south-western areas. Tropenmed. Parasitol. 30, 345–362. 575581

[64] Garms R Cheke RA Sachs R 1991 A temporary focus of savanna species of the *Simulium damnosum* complex in the forest zone of Liberia. Trop. Med. Parasitol. 42, 181–187. 1801141

[65] Wilson MD 2002 Deforestation and the spatio-temporal distribution of savannah and forest members of the *Simulium damnosum* complex in southern Ghana and south-western Togo. Trans. R. Soc. Trop. Med. Hyg. 96, 632–639. (10.1016/S0035-9203(02)90335-4) 12625139

[66] Basáñez MG McCarthy JS French MD Yang GJ Walker M Gambhir M Prichard RK Churcher TS 2012 A research agenda for helminth diseases of humans: modelling for control and elimination. PLoS Negl. Trop. Dis. 6, e1548 (10.1371/journal.pntd.0001548) 22545162 PMC3335861

[67] World Health Organization. 2012 *Accelerating work to overcome the global impact of neglected tropical diseases—a roadmap for implementation*. See http://www.who.int/neglected_diseases/NTD_RoadMap_2012_Fullversion.pdf (accessed 22 February 2014).

