# Supplementary material for: Potential effects of warmer worms and vectors on onchocerciasis transmission in West Africa
Source: Philos Trans R Soc Lond B Biol Sci. 2015 Apr 5;370(1665):20130559. doi: 10.1098/rstb.2013.0559 (PMC4342963; doi:10.1098/rstb.2013.0559)
Supplement: Supplement 1 [file rstb20130559supp1.docx]

**Supplementary Table S.1**. The development times of *Onchocerca volvulus* in various *Simulium* spp*.* at different temperatures.

| ***Simulium spp.*** | **Locality** | **Temperature Range^‡^ (°C)** | **Mean Temp (°C)** | **Duration of development (days)** | **Reference^§^** |
| --- | --- | --- | --- | --- | --- |
| *S. ochraceum* s.l. | Guatemala | 10 – 25 | 16.3 | 13.0 | 11 |
| *S. ochraceum* s.l. | Guatemala | 12 – 25 | 17.4 | 17.0 | 11 |
| *S. woodi* | Tanzania | - | 18.0 | 16.0 | 28 |
| *S. ochraceum* s.l. | Guatemala | 14 – 25 | 18.6 | 13.0 | 11 |
| *S. damnosum* s.l. | Burkina Faso | 12 – 26 | 19.0 | 10.8 | 10 |
| *S. ochraceum* s.l. | Guatemala | 16.1 – 25.6 | 19.4 | 8.0 | S1 |
| *S. metallicum* | Guatemala | 16.1 – 25.6 | 19.4 | 9.0 | S1 |
| *S. ochraceum* s.l. | Guatemala | 16 – 25 | 19.8 | 13.0 | 11 |
| *S. ochraceum* s.l. | Guatemala | 18 – 25 | 20.9 | 10.0 | 11 |
| *S. ochraceum* s.l. | Guatemala | - | 20.0 | 12.0 | S2 |
| *S. woodi* | Tanzania | - | 20.0 | 12.0 | 28 |
| *S. guianense* s.l. | Venezuela | 16 – 24.9 | 20.0 | 9.3 | 33* |
| *S. neavei* s.l. | Uganda | - | 21.0 | 6.5 | S3 |
| *S. damnosum* s.l. | Cameroon | 19.5 – 23.5 | 21.5 | 7.9 | 38* |
| *S. woodi* | Tanzania | - | 22.0 | 8.0 | 28 |
| *S. haematopotum* | Guatemala | - | 22.0 | 8.5 | S4 |
| *S. ochraceum* s.l. | Guatemala | - | 22.0 | 8.0 | 11 |
| *S. soubrense & S. sanctipauli* | Cote D’Ivoire | 19 – 25 | 22.0 | 6.5 | S5 |
| *S. ochraceum* s.l. | Guatemala | 20 – 26 | 23.0 | 8.0 | S6 |
| *S. damnosum* s.l. | Cameroon | - | 23.6 | 6.0 | S7 |
| *S. woodi* | Tanzania | - | 24.0 | 6.0 | 28 |
| *S. soubrense & S. sanctipauli* | Cote D’Ivoire | 18 – 30 | 24.0 | 6.5 | S5 |
| *S. soubrense & S. sanctipauli* | Cote D’Ivoire | 14 – 34 | 24.0 | 6.0 | S5 |
| *S. ochraceum* s.l*.* | Guatemala | 22 – 27 | 24.5 | 7.0 | S8 |
| *S. damnosum* s.l. | Tanzania | 23 – 26 | 24.5 | 6.0 | S9 |
| *S. ochraceum* s.l. | Guatemala | 24 – 26 | 25.0 | 7.7 | 31* |
| *S. damnosum* s.l. | Burkina Faso | 24 – 26 | 25.0 | 7.0 | 10 |
| *S. guianense* s.l*.* | Venezuela | 22 – 28 | 25.0 | 7.2 | 36* |
| *S. metallicum* | Venezuela | 22 – 28 | 25.0 | 5.5 | S10 |
| *S. ochraceum* s.l. | Guatemala | - | 25.0 | 6.0 | 11 |
| *S. damnosum* s.l. | Burkina Faso | 25 – 26 | 25.5 | 7.0 | 10 |
| *S. damnosum* s.l*.* | Burkina Faso | 24 – 27 | 25.5 | 6.8 | 10 |
| *S. damnosum* s.l. | Burkina Faso | 24 – 27 | 25.5 | 6.3 | 10 |
| *S. oyapockense* | Brazil | 24 – 27 | 25.5 | 7.0 | S11 |
| *S. damnosum* s.l. | Burkina Faso | 25 – 27 | 26.0 | 6.5 | 10 |
| *S. oyapockense* | Brazil | 24 – 28 | 26.0 | 7.0 | S11 |
| *S. limbatum/S. incrustatum* | Brazil | 24 – 28 | 26.0 | 6.0 | S11 |
| *S. damnosum* s.l*.* | Guinea? | 25 – 27 | 26.0 | 6.5 | S12 |
| *S. woodi* | Tanzania | - | 26.0 | 5.0 | 28 |
| *S. damnosum* s.l. | Burkina Faso | 26 – 27 | 26.5 | 6.8 | 10 |
| *S. metallicum* | Venezuela | - | 27.0 | 9.0 | 40 |
| *S. metallicum* | Venezuela | 26 – 28 | 27.0 | 6.0 | S13 |
| *S. damnosum* s.l. | Nigeria | 26 – 28 | 27.0 | 6.0 | 32 |
| *S. damnosum* s.l*.* | Burkina Faso | 26 – 28 | 27.0 | 6.8 | 10 |
| *S. exiguum* s.l. | Ecuador | 25 – 30 | 27.5 | 6.7 | 35* |
| *S. woodi* | Tanzania | - | 28.0 | 4.0 | 28 |
| *S. ochraceum* s.l. | Guatemala | - | 28.0 | 4.0 | 11 |
| *S. oyapockense & S. incrustatum* | Venezuela | 27 – 30 | 28.5 | 5.8 | 42 * |
| *S. exiguum* s.l. | Ecuador | 29 – 32 | 30.5 | 5.0 | S14 |

**^‡^** Values are omitted from the temperature range in those cases when the temperature was kept constant or only a mean was quoted by the author.

**^§^** Reference numbers refer to the list in the main text, except those preceded by S, for which see below in this supplementary file.

*Estimated by maximum likelihood as described in the main text.

**References**

[S1] Collins RC. 1979 Development of *Onchocerca volvulus* in *Simulium ochraceum* and *Simulium metallicum*. *Am. J. Trop. Med. Hyg*. **28**, 491–495.

[S2] Gibson CL. 1965 Experimental studies on the parasite and vectors of onchocerciasis in Guatemala. *Bol. Sanit. Guatemala* **36**, 246–287.

[S3] Nelson GS, Pester FR.1962 The identification of infective filarial larvae in Simuliidae. *Bull. World Health Organ.* **27**, 473–481.

[S4] Takaoka H, Suzuki H, Noda S, Ochoa A. JO, Tada I. 1984 The intake, migration and development of *Onchocerca volvulus* microfilariae in *Simulium haematopotum* in Guatemala. *Jap. J. Sanit. Zool.* **35**, 121–127.

[S5] Prod'hon J, Hebrard G, Prud’hom JM, Couret D. 1983 Etude de la capacité vectrice expérimentale de *Simulium soubrense – Simulium sanctipauli* en zone de savane humide (Région de Touba, Côte d’Ivoire). Unpublished report, O.C.C.G.E. / O.R.S.T.O.M., Bouake, Côte d’Ivoire. Available at <http://horizon.documentation.ird.fr/exl-doc/pleins_textes/pleins_textes_5/b_fdi_02-03/02701.pdf> (accessed 5th May 2014).

[S6] Collins RC, Campbell CC, Wilton DP, Newton L. 1977 Quantitative aspects of the infection of *Simulium ochraceum* by *Onchocerca volvulus. Tropenmed. Parasitol*. **28**, 235–243.

[S7] Duke BOL. 1968 Studies on factors influencing the transmission of onchocerciasis. V. The stages of *Onchocerca volvulus* in wild "forest" *Simulium dannosum,* the fate of the parasites in the fly, and the age-distribution of the biting population. *Ann. Trop. Med. Parasitol.* **62**, 107–116.

[S8] De Leon R, Duke BOL. 1966 Experimental studies on the transmission of Guatemalan and West African strains of *Onchocerca volvulus* by *Simulium ochraceum, S. metallicum* and *S. callidum. Trans. R. Soc. Trop. Med. Hyg*. **60**, 735–752.

[S9] Wegesa P. 1968 Field observations on the effect of temperature on the transmission of onchocerciasis. *Ann. Rep. East Afr. Inst. Mal. Vect. Dis.* p. 37.

[S10] Takaoka H, Suzuki H, Noda S, Tada I, Rodulfo S, Pachano L, Convit J. 1984 Susceptibility of *Simulium metallicum* to infection with *Onchocerca volvulus* in Venezuela. *Jap. J. Trop. Med. Hyg.* **12**, 89–96. (doi: http://dx.doi.org/10.2149/tmh1973.12.89)

[S11] Shelley AJ, Dias AP, Moraes MA, Procunier WS. 1987 The status of *Simulium oyapockense* and *S. limbatum* as vectors of human onchocerciasis in Brazilian Amazonia. *Med. Vet. Entomol.* **1**, 219–234. (doi: 10.1111/j.1365-2915.1987.tb00348x)

[S12] Bain O. 1969 [Morphology of the larval stages of *Onchocerca volvulus* in *Simulium damnosum* and redescription of the microfilaria]. *Ann. Parasitol. Hum. Comp.* **44**, 69–82 (article in French).

[S13] Nelson G. 1991 Human onchocerciasis: notes on the history, the parasite and the life cycle. *Ann. Trop. Med. Parasitol*. **85**, 83–95.

[S14] Collins RC, Lehmann T, Vieira Garcia JC, Guderian RH. 1995 Vector competence of *Simulium exiguum* for *Onchocerca volvulus*: implications for the epidemiology of onchocerciasis. *Am. J. Trop. Med. Hyg*. **52**, 213–218.
