# Supplementary material for: Potential effects of warmer worms and vectors on onchocerciasis transmission in West Africa
Source: Philos Trans R Soc Lond B Biol Sci. 2015 Apr 5;370(1665):20130559. doi: 10.1098/rstb.2013.0559 (PMC4342963; doi:10.1098/rstb.2013.0559)
Supplement: Supplement 2 [file rstb20130559supp2.pdf]

Cheke, R.A. *et al.* 2015. Potential effects of warmer worms and vectors on onchocerciasis transmission in West Africa. *Phil. Trans. R. Soc. B* 370 doi: 10.1098/rstb.2013.0559

Supplementary Text S.2. Derivation of the expressions for the carrying capacity (*K*) in relation to vector abundance at equilibrium, and the basic reproduction number for the vector population.

Relationship between carrying capacity and equilibrium number of flies

The following equations are as those in the main text but omit the time and temperature dependencies to simplify notation:

$$\frac{dE}{dt} = \beta_N N + \beta_P \Psi - \frac{E}{\Delta_E} - \mu_E^0\left(1 + \frac{E}{K}\right)E \qquad \text{(S.1)}$$

$$\frac{dL}{dt} = \frac{E}{\Delta_E} - \frac{L}{\Delta_L} - \mu_L L \qquad \text{(S.2)}$$

$$\frac{dP}{dt} = \frac{L}{\Delta_L} - \frac{P}{\Delta_P} - \mu_P P \qquad \text{(S.3)}$$

$$\frac{dN}{dt} = \frac{0.5P}{\Delta_P} - \left[\left(\frac{1}{g}\right) + \mu_V\right]N \qquad \text{(S.4)}$$

$$\frac{d\Psi}{dt} = \left(\frac{1}{g}\right)N - \mu_V \Psi \qquad \text{(S.5)}$$

Solve at equilibrium by setting $\frac{dE}{dt} = \frac{dL}{dt} = \frac{dP}{dt} = \frac{dN}{dt} = \frac{d\Psi}{dt} = 0$ to get steady state solutions $\left(E^*, L^*, P^*, N^*, \Psi^*\right)$ as follows:

$$\frac{d\Psi}{dt} = 0 \Rightarrow N^* = g\,\mu_V\,\Psi^* \qquad \text{(S.6)}$$

$$\frac{dN}{dt} = 0 \Rightarrow P^* = 2\Delta_P\left(\mu_V + g\,\mu_V{}^2\right)\Psi^* \qquad \text{(S.7)}$$

$$\frac{dP}{dt} = 0 \Rightarrow L^* = 2\Delta_L (1 + \mu_P \Delta_P)\left(\mu_V + g\,\mu_V{}^2\right)\Psi^* \qquad \text{(S.8)}$$

$$\frac{dL}{dt} = 0 \Rightarrow E^* = 2\Delta_E (1 + \mu_L \Delta_L)(1 + \mu_P \Delta_P)\left(\mu_V + g\,\mu_V{}^2\right)\Psi^* \qquad \text{(S.9)}$$

Set $\omega = 2\Delta_E (1+ \mu_L \Delta_L)(1+ \mu_P \Delta_P)\left(\mu_V + g\,\mu_V{}^2\right)$. Now solve $\frac{dE}{dt} = 0$ and re-arrange to get the relationship between K and the equilibrium number of flies.

$$\beta_N g \mu_V \Psi^* + \beta_P \Psi^* - \frac{\omega}{\Delta_E}\Psi^* - \mu_E^0 \left(1 + \frac{\omega \Psi^*}{K}\right)\omega \Psi^* = 0$$

$$\Psi^* = K\left[\frac{\beta_N g \mu_V + \beta_P - \left(\frac{\omega}{\Delta_E}\right) - \mu_E^0 \omega}{\mu_E^0 \omega^2}\right] \qquad \text{(S.10)}$$

$$N^* = K\left\{\frac{g\mu_V\left[\beta_N g \mu_V + \beta_P - \left(\frac{\omega}{\Delta_E}\right) - \mu_E^0 \omega\right]}{\mu_E^0 \omega^2}\right\} \qquad \text{(S.11)}$$

$$V^* = N^* + \Psi^* = K\left\{\frac{(1 + g\mu_V)\left[\beta_N g \mu_V + \beta_P - \left(\frac{\omega}{\Delta_E}\right) - \mu_E^0 \omega\right]}{\mu_E^0 \omega^2}\right\} \qquad \text{(S.12)}$$

For a given baseline egg mortality μ_E^0 we can calculate the egg mortality at equilibrium density $\mu_E(E^*)$ (i.e. after the excess mortality due to density dependence has taken place) as follows:

$$\mu_E(E^*) = \mu_E^0\left(1+\frac{E^*}{K}\right) = \mu_E^0\left(1+\frac{\omega\,\Psi^*}{K}\right) = \mu_E^0\left\{1+\omega\left[\frac{\beta_N g\mu_V + \beta_P - \left(\frac{\omega}{\Delta_E}\right) - \mu_E^0\omega}{\mu_E^0\omega^2}\right]\right\} = \frac{\beta_N g\mu_V}{\omega} + \frac{\beta_P}{\omega} - \frac{1}{\Delta_E}$$

(S.13)

This expression is independent of μ_E^0 so that no matter how low the baseline mortality is, $\mu_E(E^*)$ will rise to the same level after density dependent regulation takes effect.

Blackfly Reproduction Number

The basic reproduction number of the blackfly vector population (R_0^{BF}) is the number of adult female flies produced by a female fly during her lifespan in the absence of density dependent constraints. It is possible to derive this expression empirically as follows:

$$R_0^{BF} = \left(\frac{1}{2}\right)\left(\frac{\beta_N}{1/g+\mu_V} + \frac{1}{1+\mu_V g}\frac{\beta_P}{\mu_V}\right)\left(\frac{1}{1+\mu_E^0\Delta_E}\right)\left(\frac{1}{1+\mu_L\Delta_L}\right)\left(\frac{1}{1+\mu_P\Delta_P}\right) \quad \text{(S.14)}$$

where half of the flies are female; $\left(\frac{\beta_N}{1/g+\mu_V}\right)$ is the number of eggs laid by nulliparous flies; $\left(\frac{1}{1+\mu_V g}\right)$ is the proportion of nulliparous flies that survive to become parous; $\left(\frac{\beta_P}{\mu_V}\right)$ is the number of eggs laid by parous flies; $\left(\frac{1}{1+\mu_E^0\Delta_E}\right)$ is the proportion surviving the egg stage; $\left(\frac{1}{1+\mu_L\Delta_L}\right)$ is the proportion surviving the larval stage and $\left(\frac{1}{1+\mu_P\Delta_P}\right)$ is the proportion surviving the pupal stage to become adult flies.
