# Supplementary material for: Potential effects of warmer worms and vectors on onchocerciasis transmission in West Africa
Source: Philos Trans R Soc Lond B Biol Sci. 2015 Apr 5;370(1665):20130559. doi: 10.1098/rstb.2013.0559 (PMC4342963; doi:10.1098/rstb.2013.0559)
Supplement: Supplement 3 [file rstb20130559supp3.docx]

**Supplementary Text S.3. Stability analysis of the blackfly population model based on the formula for the basic reproduction number derived in Supplementary text 2**

By using the next generation matrix approach [S15], the basic reproduction number of the blackfly vector population $R_{0}^{BF}$ was derived as follows:

.

**Theorem 1** *If $R_{0}^{BF}\leq1$, then the no-flies steady state* $\overline{X}(0,0,0,0,0)$*of the system is globally asymptotically stable and unstable otherwise.*

***Proof*** Construct a continuously differentiable and non-negative Lyapunov function

$$V=E+c_{1}L+c_{2}P+c_{3}N+c_{4}\Psi$$

where

$c_{1}=$ ,

(S3.1)

$\frac{\beta_{P}}{\mu_{V}}$,

then we obtain

$$\frac{dV}{dt}=\frac{dE}{dt}+c_{1}\frac{dL}{dt}+c_{2}\frac{dP}{dt}+c_{3}\frac{dN}{dt}+c_{4}\frac{d\Psi}{dt}$$

$$=\left[ \beta_{N}N+\beta_{P}\Psi-\frac{E}{\Delta_{E}}-\mu_{E}^{0}\left( 1+\frac{E}{K} \right)E \right]+c_{1}\left( \frac{E}{\Delta_{E}}-\frac{L}{\Delta_{L}}-\mu_{L}L \right)+c_{2}\left( \frac{L}{\Delta_{L}}-\frac{P}{\Delta_{P}}-\mu_{P}P \right)$$

$+c_{3}\left[ \frac{0.5P}{\Delta_{P}}-\left( \frac{1}{g}+\mu_{V} \right)N \right]+c_{4}\left( \frac{1}{g}N-\mu_{V}\Psi\right)$

$$=E\left[ \frac{c_{1}}{\Delta_{E}}-\left( \frac{1}{\Delta_{E}}+\mu_{E}^{0} \right)-\frac{\mu_{E}^{0}E}{K} \right]+ L\left[ \frac{c_{2}}{\Delta_{L}}-c_{1}\left( \frac{1}{\Delta_{L}}+\mu_{L} \right) \right]+P\left[ \frac{c_{3}}{2\Delta_{P}}-c_{2}\left( \frac{1}{\Delta_{P}}+\mu_{P} \right) \right]$$

$+N\left[ \frac{c_{4}}{g}+\beta_{N}-c_{3}\left( \frac{1}{g}+\mu_{V} \right) \right]+\Psi\left( \beta_{P}-c_{4}\mu_{V} \right)$

$=\frac{E}{\Delta_{E}}\left( \frac{1}{1+\mu_{E}^{0}\Delta_{E}} \right)\left( R_{0}^{BF}-1 \right)-\frac{\mu_{E}^{0}}{K}E^{2}$ ≤ 0.

The equality $\frac{dV}{dt}=0$ holds if and only if *E* = 0. Then we get *L* = *P* = *N* = *Ψ* = 0 from the ODE model. Thus, by the LaSalle largest invariant set theorem, the no-flies steady state

$\bar{X}$ (0,0,0,0,0) is globally asymptotically stable when $R_{0}^{BF}\leq1$. In this case, the flies go extinct.

Next, we prove that $\bar{X}$ is unstable when $R_{0}^{BF}>1$. The Jacobian matrix $J\left( \bar{X} \right)$ of the system at $\bar{X}$ is

$J\left(\bar{X}\right)=\left(\begin{matrix} -\left(\frac{1}{\text{Δ}_{E}}+\mu_{E}^{0}\right) 0 0 \beta_{N} \beta_{P} \\ \frac{1}{\text{Δ}_{E}} - \left(\frac{1}{\text{Δ}_{L}}+\mu_{L}\right) 0 0 0 \\ 0 \frac{1}{\text{Δ}_{L}} - \left(\frac{1}{\text{Δ}_{P}}+\mu_{P}\right) 0 0 \\ 0 0 \frac{1}{\text{2Δ}_{P}} - \left(\frac{1}{g}+\mu_{V}\right) 0 \\ 0 0 0 \frac{1}{g} -\mu_{V} \end{matrix}\right)$.

For convenience of notation, let

$$h_{1}=\frac{1}{\text{Δ}_{E}}+\mu_{E}^{0},h_{2}=\frac{1}{\text{Δ}_{L}}+\mu_{L},h_{3}=\frac{1}{\text{Δ}_{P}}+\mu_{P},h_{4}=\frac{1}{g}+\mu_{V},h_{5}=\mu_{V}.$$

Then we obtain the characteristic equation at $\bar{X}$ as follows:

$\left( \lambda+h_{1} \right)\left( \lambda+h_{2} \right)\left( \lambda+h_{3} \right)\left( \lambda+h_{4} \right)\left( \lambda+h_{5} \right)-\beta_{N}\frac{1}{\Delta_{E}}\frac{1}{\Delta_{L}}\frac{1}{2\Delta_{P}}\left( \lambda+h_{5} \right)-\beta_{P}\frac{1}{\Delta_{E}}\frac{1}{\Delta_{L}}\frac{1}{2\Delta_{P}}\frac{1}{g}$ $=\lambda^{5}+k_{1}\lambda^{4}+k_{2}\lambda^{3}+k_{3}\lambda^{2}+k_{4}\lambda+k_{5}=0,$ (S3.2)

where

*k_1_* = *h_1_ + h_2_ + h_3_ + h_4_ + h_5，_*

*k_2_* = *h_1_* (*h_2_ + h_3_ + h_4_ + h_5_*) + *h_2_*(*h_3_ + h_4_ + h_5_*) + *h_3_* (*h_4_ + h_5_*) + *h_4_h_5_,*

*k_3_ = h_1_* (*h_2_h_3_ + h_2_h_4_ + h_2_h_5_ + h_3_h_4_ + h_3_h_5_ + h_4_h_5_*) + *h_2_*(*h_3_h_4_ + h_3_h_5_ + h_4_h_5_*) + *h_3_h_4_h_5_,*

*k_4_ = h_1_* (*h_2_h_3_h_4_ + h_2_h_3_h_5_ + h_2_h_4_h_5_ + h_3_h_4_h_5_*) *+ h_2_h_3_h_4_h_5_* ${-\beta}_{N}\frac{1}{\Delta_{E}}\frac{1}{\Delta_{L}}\frac{1}{2\Delta_{P}}$,

*k_5_* = *h_1_h_2_h_3_h_4_h_5_* $-\beta_{N}\frac{1}{\Delta_{E}}\frac{1}{\Delta_{L}}\frac{1}{2\Delta_{P}}h_{5}-\beta_{P}\frac{1}{\Delta_{E}}\frac{1}{\Delta_{L}}\frac{1}{2\Delta_{P}}\frac{1}{g}$ .

Denote the eigenvalues of the characteristic equation (S3.2) as λ_i_ , i = 1,2,...5. When $R_{0}^{BF}>1$, we get

$k_{5}=\left( \frac{1}{\Delta_{E}}+\mu_{E}^{0} \right)\left( \frac{1}{\Delta_{L}}+\mu_{L} \right)\left( \frac{1}{\Delta_{P}}+\mu_{P} \right)\left( \frac{1}{g}+\mu_{V} \right)\mu_{V}-$

$\beta_{N}\frac{1}{\Delta_{E}}\frac{1}{\Delta_{L}}\frac{1}{2\Delta_{P}}\mu_{V}-\beta_{P}\frac{1}{\Delta_{E}}\frac{1}{\Delta_{L}}\frac{1}{2\Delta_{P}}\frac{1}{g}$

=$\left( \frac{1}{\Delta_{E}}+\mu_{E}^{0} \right)\left( \frac{1}{\Delta_{L}}+\mu_{L} \right)\left( \frac{1}{\Delta_{P}}+\mu_{p} \right)\left( \frac{1}{g}+\mu_{V} \right)\mu_{V}\left( 1-R_{0}^{BF} \right)$

$<0$,

which means that $\lambda_{1}\lambda_{2}\lambda_{3}\lambda_{4}\lambda_{5}=-k_{5}>0.$ This indicates that at least one of $\lambda_{1},\lambda_{2},\lambda_{3},\lambda_{4},\lambda_{5}$ has a positive real part. Thus, $\bar{X}$ is unstable if $R_{0}^{BF}>1$. This completes the proof.

**Theorem 2** *When $R_{0}^{BF}>1$, then the unique positive equilibrium is globally asymptotically stable.*

***Proof*** Construct a continuously differentiable and non-negative Lyapunov function

$$W=E-E^{*}-E^{*}\ln\frac{E}{E^{*}}+c_{1}\left( L-L^{*}-L^{*}\ln\frac{L}{L^{*}} \right)+c_{2}\left( P-P^{*}-P^{*}\ln\frac{P}{P^{*}} \right)$$

$+c_{3}\left( N-N^{*}-N^{*}\ln\frac{N}{N^{*}} \right)+c_{4}\left( \Psi-\Psi^{*}-\Psi^{*}\ln\frac{\Psi}{\Psi^{*}} \right)$ ,

where $c_{1},c_{2},$ $c_{3},c_{4}$ are given by equation S3.1. The global minimum of *W* occurs at the positive equilibrium , and the function *W* takes the value *W* = 0 at the positive equilibrium .

Differentiating *W* along the solutions of the system and using the equilibrium relations and equation S3.1, we obtain

$$\frac{dW}{dt}=\left( 1-\frac{E}{E^{*}} \right)\left[ \beta_{N}N+\beta_{P}\Psi-\frac{E}{\Delta_{E}}-\mu_{E}^{0}\left( 1+\frac{E}{K} \right)E \right]+c_{1}\left( 1-\frac{L^{*}}{L} \right)\left( \frac{E}{\Delta_{E}}-\frac{L}{\Delta_{L}}-\mu_{L}L \right)$$

$$+c_{2}\left( 1-\frac{P^{*}}{P} \right)\left( \frac{L}{\Delta_{L}}-\frac{P}{\Delta_{P}}-\mu_{P}P \right)+c_{3}\left( 1-\frac{N^{*}}{N} \right)\left[ \frac{0.5P}{\Delta_{P}}-\left( \frac{1}{g}+\mu_{V} \right)N \right]$$

$+c_{4}\left( 1-\frac{\Psi^{*}}{\Psi} \right)\left( \frac{1}{g}N-\mu_{V}\Psi\right)$

$=$

$\beta_{N}N+\beta_{P}\Psi-\left( \frac{1}{\Delta_{E}}+\mu_{E}^{0} \right)E-\frac{\mu_{E}^{0}}{K}E^{2}-\beta_{N}E^{*}\frac{N}{E}-\beta_{P}E^{*}\frac{\Psi}{E}+\left( \frac{1}{\Delta_{E}}+\mu_{E}^{0} \right)E^{*}+\frac{\mu_{E}^{0}E^{*}}{K}E$

$+c_{1}\left[ \frac{E}{\Delta_{E}}-\left( \frac{1}{\Delta_{L}}+\mu_{L} \right)L-\frac{L^{*}}{\Delta_{E}}\frac{E}{L}+\left( \frac{1}{\Delta_{L}}+\mu_{L} \right)L^{*} \right]$

$+c_{2}\left[ \frac{L}{\Delta_{L}}-\left( \frac{1}{\Delta_{P}}+\mu_{P} \right)P-\frac{P^{*}}{\Delta_{L}}\frac{L}{P}+\left( \frac{1}{\Delta_{P}}+\mu_{P} \right)P^{*} \right]$

$+c_{3}\left[ \frac{P}{{2\Delta}_{P}}-\left( \frac{1}{g}+\mu_{V} \right)N-\frac{N^{*}}{{2\Delta}_{P}}\frac{P}{N}+\left( \frac{1}{g}+\mu_{V} \right)N^{*} \right]$

$+c_{4}\left( \frac{1}{g}N-\mu_{V}\Psi-\frac{\Psi^{*}}{g}\frac{N}{\Psi}+\mu_{V}\Psi^{*} \right)$

$=E\left[ \frac{c_{1}}{\Delta_{E}}-\left( \frac{1}{\Delta_{E}}+\mu_{E}^{0} \right)+\frac{\mu_{E}^{0}E^{*}}{K} \right]-\frac{\mu_{E}^{0}}{K}E^{2}+L\left[ \frac{c_{2}}{\Delta_{L}}-c_{1}\left( \frac{1}{\Delta_{L}}+\mu_{L} \right) \right]$

$+P\left[ \frac{c_{3}}{2\Delta_{P}}-c_{2}\left( \frac{1}{\Delta_{P}}+\mu_{P} \right) \right]+N\left[ \frac{c_{4}}{g}+\beta_{N}-c_{3}\left( \frac{1}{g}+\mu_{V} \right) \right]+\Psi\left( \beta_{P}-c_{4}\mu_{V} \right)$

$-\beta_{N}E^{*}\frac{N}{E}-$

$\beta_{P}E^{*}\frac{\Psi}{E}-c_{1}\frac{L^{*}}{\Delta_{E}}\frac{E}{L}-c_{2}\frac{P^{*}}{\Delta_{L}}\frac{L}{P}-c_{3}\frac{N^{*}}{2\Delta_{P}}\frac{P}{N}-c_{4}\frac{\Psi^{*}}{g}\frac{N}{\Psi}$

$+\left( \frac{1}{\Delta_{E}}+\mu_{E}^{0} \right)E^{*}+c_{1}\left( \frac{1}{\Delta_{L}}+\mu_{L} \right)L^{*}+c_{2}\left( \frac{1}{\Delta_{P}}+\mu_{P} \right)P^{*}+$

$c_{3}\left( \frac{1}{g}+\mu_{V} \right)N^{*}+ c_{4}\mu_{V}\Psi^{*}$

$=-\frac{\mu_{E}^{0}}{K}\left[ E-\frac{K}{\mu_{E}^{0}}\left( \frac{1}{\Delta_{E}}+\mu_{E}^{0} \right)\left( R_{0}^{BF}-1 \right) \right]^{2}-\beta_{N}N^{*}\left( \frac{E^{*}}{N^{*}}\frac{N}{E}+\frac{L^{*}}{E^{*}}\frac{E}{L}+\frac{P^{*}}{L^{*}}\frac{L}{P}+\frac{N^{*}}{P^{*}}\frac{P}{N}-4 \right)$

$-$

$\beta_{P}\Psi^{*}\left( \frac{E^{*}}{\Psi^{*}}\frac{\Psi}{E}+\frac{L^{*}}{E^{*}}\frac{E}{L}+\frac{P^{*}}{L^{*}}\frac{L}{P}+\frac{N^{*}}{P^{*}}\frac{P}{N}+\frac{\Psi^{*}}{N^{*}}\frac{N}{\Psi}-5 \right).$

Because the geometric mean is always less than or equal to the arithmetic mean, we have and the equality holds if and only if (*E*, *L*, *P*, *N*, *Ψ*) take the equilibrium values

${(E}^{*}, L^{*}, P^{*}, N^{*}, \Psi^{*}).$ Therefore, by the LaSalle largest invariant set theorem, it follows that the positive equilibrium is globally asymptotically stable.

Reference

[S15] van den Driessche P, Watmough, J. 2002 Reproduction numbers and sub-threshold endemic equilibria for compartmental models of disease transmission. *Mathematical Biosciences* **180**, 29-48. ([doi:10.1016/S0025-5564(02)00108-6](http://dx.doi.org/10.1016/S0025-5564%2802%2900108-6))
